# Personalized chronotherapy in glioblastoma: integrating circadian profiling and PK–PD modelling to optimize temozolomide timing

**DOI:** 10.1038/s41698-025-01205-z

**Published:** 2025-12-12

**Authors:** Nina Nelson, Oliver Zimmer, Angela Relógio

**Affiliations:** 1 Institute for Systems Medicine and Faculty of Human Medicine, MSH Medical School Hamburg, Hamburg, Germany; 2 TimeTeller GmbH, 22085 Hamburg, Germany

**Keywords:** Cancer, Computational biology and bioinformatics, Neuroscience, Oncology

## Abstract

Glioblastoma (GBM) remains one of the most lethal brain tumors, with limited benefit from current standard therapies, including temozolomide (TMZ). Chronotherapy—aligning treatment with the circadian clock—has shown improved cancer outcomes, but its clinical efficacy in GBM remains inconsistent, potentially due to a lack of personalization. Here, we present an integrated experimental and computational approach to investigate personalized TMZ chronotherapy. Using a GBM in vitro model, along with genetic and pharmacological manipulation of clock genes (*BMAL1, NR1D1, PER2*), we show that TMZ sensitivity is time-of-day dependent. Clock gene disruption reduced TMZ efficacy, likely through altered DNA repair regulation. Co-treatment with clock modulators modulated TMZ response dependent on clock phenotype. A mechanistic pharmacokinetic–pharmacodynamic model incorporating the clock network recapitulated experimental observations and enabled prediction of treatment timing. Our findings highlight the importance of timing in GBM therapy and propose combining circadian profiling with mathematical modeling to personalize GBM chronotherapy.

## Introduction

Glioblastoma (GBM) remains one of the most aggressive primary brain tumors in adults. Despite decades of research, prognosis remains poor, with median survival reported to be less than 15 months^1^ and approximately 90% of patients experiencing relapse following first-line therapy^2^. Although GBM is relatively rare, affecting approximately 3.19–4.17 individuals per 100,000 annually, it accounts for nearly half of all primary brain tumors and often causes severe neurological symptoms that significantly impair the quality of life of both patients and their caregivers^1^.

The current standard-of-care, known as the Stupp protocol, was established in 2005 and has yet to be surpassed despite suboptimal outcomes^3^. This regimen consists of two phases: an initial phase of radiotherapy (5 days per week) combined with daily temozolomide (TMZ) chemotherapy for 6 weeks, followed by a maintenance phase of six 28-day cycles of TMZ (administered on days 1–5 of each cycle).

Chronotherapy—an approach that aligns treatment administration with the patient’s circadian rhythm, or pharmacologically modulates the circadian clock in conjunction with anti-cancer therapy^4^—has shown potential to enhance treatment efficacy in several cancer types^5–7^. In preclinical GBM models, circadian rhythms have been observed in both core-clock genes and MGMT, a DNA repair enzyme implicated in resistance to TMZ. Notably, TMZ sensitivity in both human (LN229) and murine (GL261) GBM cell lines peaked during maximal *BMAL1* expression and minimal *MGMT* activity^8–10^. This finding aligns with in vivo studies reporting enhanced responses to high-dose TMZ therapy when administered in the morning^9^.

Supporting this, a retrospective clinical study involving 166 patients found improved overall survival when TMZ was administered in the morning^11^. However, results from prospective clinical trials have been inconclusive. One such trial (*n* = 35) comparing morning versus evening TMZ administration found no significant differences in adverse effects, quality of life, or survival outcomes^12^. A recent analysis of data from the CENTRIC-EORTC (MGMT-methylated, *n* = 545) and CORE (MGMT-unmethylated, *n* = 265) trials similarly reported no survival benefit, but observed increased bone marrow toxicity in patients receiving morning TMZ^13^. A pilot study involving 10 patients further suggested that, during TMZ maintenance therapy, the alignment between internal circadian time (as assessed via actigraphy) and fixed wall-clock time may be disrupted, with some patients’ internal morning corresponding more closely to evening hours on the clock^14^. These findings imply that aligning treatment with individual circadian profiles, rather than external time alone, may improve therapeutic outcomes.

To refine and personalize TMZ-based chronotherapy in GBM, it is critical to: (1) elucidate the mechanistic underpinnings of circadian modulation of drug response; (2) develop approaches to align drug administration with internal time biomarkers; and (3) validate such strategies in larger, prospective clinical studies.

Mathematical modeling offers a promising avenue for optimizing and personalizing chronotherapy. Several efforts have aimed to model circadian fluctuations in drug efficacy broadly^15–18^ and in TMZ treatment specifically^15^. However, while a few mathematical models contain a mechanistic integration of the core-clock network (CCN)^18,19^, most existing models rely on simplified harmonic representations of circadian rhythms and lack an explicit CCN.

In this study, we employed GBM in vitro models with distinct genetic backgrounds to investigate how circadian profiles influence TMZ response. We further explored pharmacological modulation of the clock using the CRY agonist KL001 and the REV-ERB agonist SR9011—agents previously shown to enhance TMZ efficacy, both alone and in combination, in GBM experimental models^20,21^. Additionally, we developed a fully mechanistic mathematical model linking the circadian clock to TMZ pharmacodynamics, which closely mirrored our experimental observations.

Our extended model enables mechanistically grounded simulations of genetic alterations (e.g., knockouts, inter-individual variations) in core-clock components and their downstream effects on TMZ efficacy. This significantly expands the predictive capacity of previously published models.

Together, our data demonstrate the potential of combining circadian clock profiling with mathematical modeling to advance personalized TMZ chronotherapy. This integrative approach holds promise for future clinical translation to improve outcomes in GBM patients.

## Results

### Circadian profiling reveals distinct rhythmic signatures across GBM cell lines

**Fig. 1:**
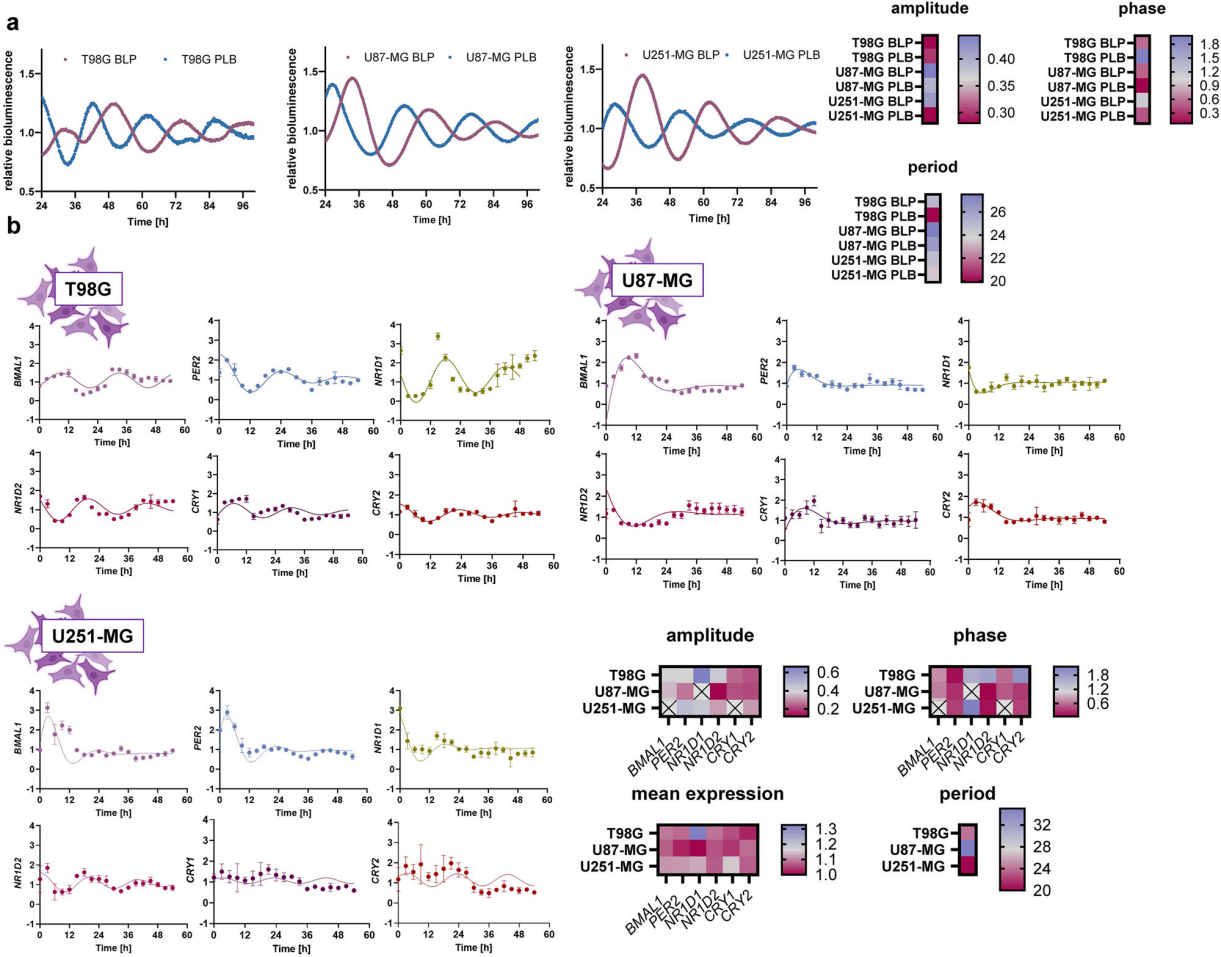
Glioblastoma cell lines differ in their circadian profile. **a** Circadian profile on the cellular level. GBM cell lines were transduced with luminescent reporters to record *BMAL1* or *PER2* promotor activity in living cells. The experiment was repeated three times with three biological replicates. Circadian parameters were calculated by Chronostar 3 software. **b** The circadian profiles of selected core-clock genes were measured by q-RT-PCR and normalized to GAPDH and the mean gene expression. The experiment was performed in three biological replicate and for each biological replicate three technical replicates were measured. Circadian parameters were determined by cosinor analysis. Error bars signify standard deviation. Crossed boxes in the heatmaps indicate no significant rhythmicity (*p* > 0.05) in the cosinor analysis. One-way ANOVA was used for statistical comparisons.

To investigate the role of circadian phenotype in GBM chronotherapy, we employed three genetically distinct GBM cell lines—U87-MG, T98G, and U251-MG—as model systems representing inter-patient variability. Given growing evidence supporting the relevance of individual circadian profiling in treatment response, we first characterized the circadian behavior of each cell line using bioluminescent reporters driven by *BMAL1* or *PER2* promoters (Fig. 1a).

All three cell lines exhibited robust circadian oscillations at the promoter level. Notably, each showed a significant phase difference between *BMAL1* and *PER2* activity: 0.4 rad in U87-MG (*p* = 0.0004), 1.5 rad in T98G (*p* = 0.0028), and 0.7 rad in U251-MG (*p* = 0.0005). U87-MG and U251-MG did not differ significantly in circadian period for either promoter (*BMAL1*: *p* = 0.21; *PER2*: *p* = 0.18). U87-MG displayed an extended period (26.9 ± 0.9 h), exceeding the canonical 24-hour cycle, while U251-MG oscillated within the normal range (24.0 ± 0.8 h). T98G cells exhibited discordant periods between promoters: *BMAL1* oscillated normally (24.8 ± 1.9 h), but *PER2* had a significantly shortened period of 19.9 ± 2.1 h (*p* = 0.0023).

Amplitude and phase comparisons further highlighted cell line-specific differences. At the *BMAL1* promoter level, T98G showed significantly lower amplitude than U87-MG (*p* = 0.0053) and U251-MG (*p* = 0.030). U87-MG had a significantly longer period than T98G (*p* = 0.012) and U251-MG (*p* = 0.0067). Phase was significantly delayed in U251-MG compared to T98G (*p* = 0.0019) and U87-MG (*p* < 0.0001), while U87-MG and T98G had comparable phases (*p* = 0.79). At the *PER2* promoter level, amplitudes did not differ significantly, but both period and phase were significantly different across all three cell lines (*period*: all comparisons *p* < 0.01; *phase*: all comparisons *p* < 0.001).

To extend these observations to the transcript level, we measured endogenous expression of six core-clock genes (*BMAL1*, *PER2*, *NR1D1*, *NR1D2*, *CRY1*, and *CRY2*) by qRT-PCR over a 54-hour period in 3-hour intervals (Fig. 1b). Expression was normalized to *GAPDH* and mean expression. The best-fit period used for Cosinor analysis was 24 h in T98G, 35 h in U87-MG, and 20 h in U251-MG.

Cosinor analysis revealed significant differences in both phase and amplitude between cell lines. T98G displayed the most robust rhythmicity, with all six core-clock genes showing significant 24-hour oscillations (*p* < 0.05) and stable amplitude across the time course. In contrast, U87-MG and U251-MG lacked rhythmicity in some genes and showed either lengthened (U87-MG) or shortened (U251-MG) periods, consistent with promoter-level observations. These disrupted rhythms likely reflect desynchronization due to imbalances in the clock network, which may underlie the observed rapid damping of oscillations in these two cell lines.

Interestingly, while *BMAL1* and *PER2* phase relationships were largely preserved between promoter activity and mRNA expression, discrepancies in rhythm robustness suggest post-transcriptional regulation plays a role in shaping the observed RNA profiles. In U87-MG and U251-MG, but less so in T98G, oscillations appeared less stable at the mRNA level than at the promoter level, possibly due to cell line-specific differences in mRNA processing, stability, or feedback regulation.

These findings underscore substantial variability in circadian clock function across GBM cell lines, supporting the hypothesis that inter-patient circadian differences may influence response to chronotherapy.

### Chronotherapeutic effects of TMZ and clock modulators differ by circadian phenotype and timing

Having established distinct circadian profiles across our GBM cell line models, we next investigated whether these differences influence responses to chronotherapy. We focused primarily on TMZ, which has been reported to exhibit time-dependent efficacy in both preclinical and clinical GBM studies^8–14^. TMZ is a well-established component of standard GBM therapy and has a short half-life (~1.8 h), making it amenable to precise timing of administration^22^.

**Fig. 2:**
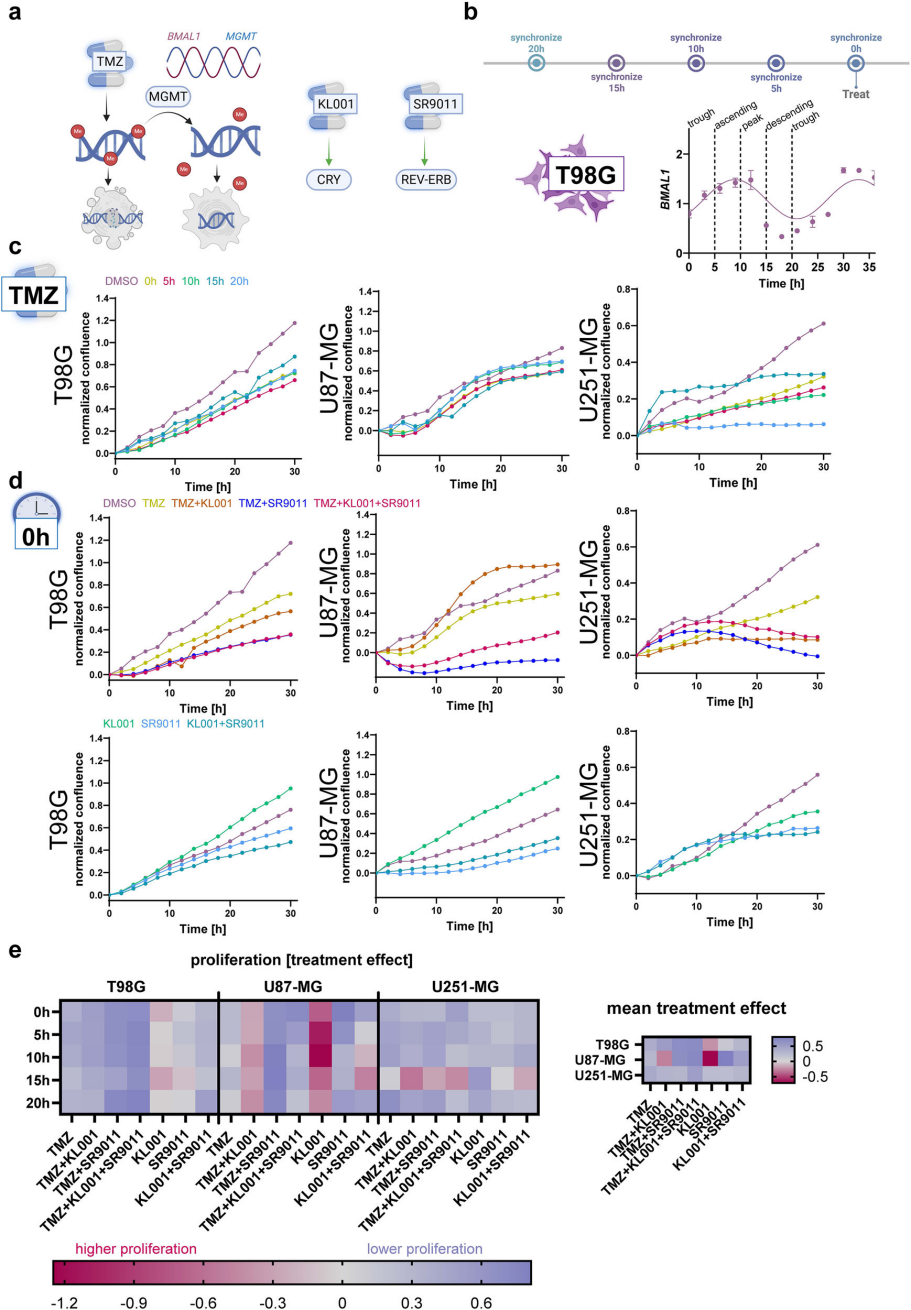
Glioblastoma cell lines show distinct time-dependent treatment responses to TMZ and clock modulators. **a** TMZ was shown to methylate the DNA leading to DNA damage and apoptosis. MGMT is able to remove methyl-adducts thus preventing DNA damage and apoptosis. BMAL1 and MGMT were shown to be antiphase and TMZ treatment sensitivity highest and the peak of BMAL1 expression. KL001 was shown to stabilize CRY. SR9011 was described as an REV-ERB (NR1D1 and NR1D2) agonist. Both drugs were chosen as they showed promising and specific effects in GBM cell lines and mouse models before. **b** Timepoints for time-dependent treatment were chosen based on distinct points in the *BMAL1* expression profile of T98G cells which showed the most robust rhythms. This was done based on previous reports suggesting a link between BMAL1 rhythm and TMZ sensitivity. The experimental scheme regarding synchronization at different timepoints prior to treatment is depicted. **c** Shows normalized proliferation curves for TMZ for the different synchronization times and **d** Shows the normalized proliferation curves for the different treatments at 0 h synchronization. Shown is the mean over nine measurements (three biological replicates with three technical replicates each). Cells were synchronized by medium change and treated with the drugs at the indicated concentrations. Proliferation measurements were performed as percent confluence in the IncuCyte S5. **e** The heatmap of treatment effects for all performed treatments. AUC was calculated and normalized to the DMSO control of the respective plate. Treatment effect was calculated as 1-AUC. Therefore, DMSO had a treatment effect of 0, cells that proliferated less than DMSO had a treatment effect >0 and cells that proliferated more than DMSO had a treatment effect<0. Statistical analysis was performed using One-way ANOVA. Error bars represent standard deviation. Icons and the scheme in panel a were generated in Biorender.com.

To further assess the influence of circadian regulation, we also evaluated two pharmacological modulators of the core-clock: the CRY agonist KL001^23^ and the REV-ERB agonist SR9011^24^. Both have demonstrated selective anti-tumor activity in GBM models and offer the ability to experimentally modulate circadian dynamics^20,21^ (Fig. 2a).

As *BMAL1* expression levels have previously been correlated with TMZ sensitivity, we selected treatment timepoints based on the circadian phase of *BMAL1* expression: 0 h (trough), 5 h (ascending), 10 h (peak), 15 h (descending), and 20 h (trough) (Fig. 2b). Cells were treated at these timepoints with TMZ, KL001, or SR9011 alone or in combination, at their respective IC_50_ concentrations (Supplementary Fig. 1). Proliferation was monitored in real time (Fig. 2c, d**;** Supplementary Figs. 2–4), and treatment efficacy was quantified as the normalized area under the curve (AUC) relative to DMSO control. The treatment effect was defined as 1 - AUC, where positive values indicate growth inhibition and negative values indicate enhanced proliferation (Supplementary Table 1). Mean treatment effects across all timepoints were also calculated for each condition (Fig. 2e).

### TMZ-Based combinations outperform clock modulators alone

In T98G cells, TMZ-based treatments were clearly more effective than those with clock modulators alone. KL001 exhibited a consistent negative treatment effect across all timepoints, while SR9011 alone showed limited efficacy. Interestingly, the combination of KL001 + SR9011 yielded a comparable effect to TMZ alone (*p* = 0.62) and was significantly more effective at 20 h (*p* = 0.0395). The most effective treatments were combinations of TMZ with one or both clock modulators. TMZ + SR9011 and TMZ + KL001 + SR9011 significantly outperformed TMZ monotherapy independent of treatment timing. These findings support the potential of clock-targeting agents to enhance TMZ efficacy in T98G cells (Fig. 2c–e; Supplementary Fig. 2, Supplementary Table 1).

### Selective benefit from SR9011 and time-specific responses

U87-MG cells exhibited a different profile. KL001 consistently induced negative treatment effects, and in combination with TMZ, appeared to antagonize its efficacy. In contrast, SR9011 showed the highest overall efficacy among monotherapies, significantly outperforming TMZ alone (*p* = 0.01).

Notably, the efficacy of SR9011 and its combinations was highly time-dependent. The most effective treatments included SR9011 at 0 h and TMZ + SR9011 at 10 h, 15 h, and 20 h. These results highlight that both the choice of agent and treatment timing are critical for maximizing efficacy in U87-MG cells (Fig. 2c–e; Supplementary Fig. 3, Supplementary Table 1).

### Circadian time-dependent sensitivity and unique KL001 response

U251-MG cells demonstrated a strong time-of-treatment effect, with the poorest responses generally occurring at 15 h. In contrast, 20 h emerged as the optimal treatment time (*p* = 0.005 vs. 15 h). The most effective single agent was TMZ at 20 h, followed by KL001 at 15 h, which was surprising given its poor performance in the other two cell lines.

Indeed, KL001 exhibited a notably different response in U251-MG cells, with a positive mean treatment effect, suggesting a potentially unique CRY-dependent regulatory mechanism in this line. This differential response may reflect underlying differences in the CRY network, given KL001’s activation of CRY^23,25^ (Fig. 2c–e; Supplementary Fig. 4, Supplementary Table 1).

Furthermore, T98G cells displayed an overall lower amplitude in treatment effect—particularly for the clock modulators—which may correlate with their lower circadian amplitude observed at the promoter level (Fig. 2e). This suggests that intrinsic clock robustness may influence responsiveness to clock-targeting chronotherapeutics.

Together, these results demonstrate that the circadian clock modulates the efficacy of TMZ and clock-targeting agents in a time- and cell line-dependent manner. Most treatments showed significant time-of-day variation, and pharmacological manipulation of the CCN altered treatment responses. Importantly, each GBM cell line, representing a distinct circadian phenotype, responded differently to both the timing and type of treatment. These findings underscore the importance of personalized chronotherapy strategies based on individual circadian profiles in GBM.

### TMZ treatment response is mechanistically coupled to the circadian clock

**Fig. 3:**
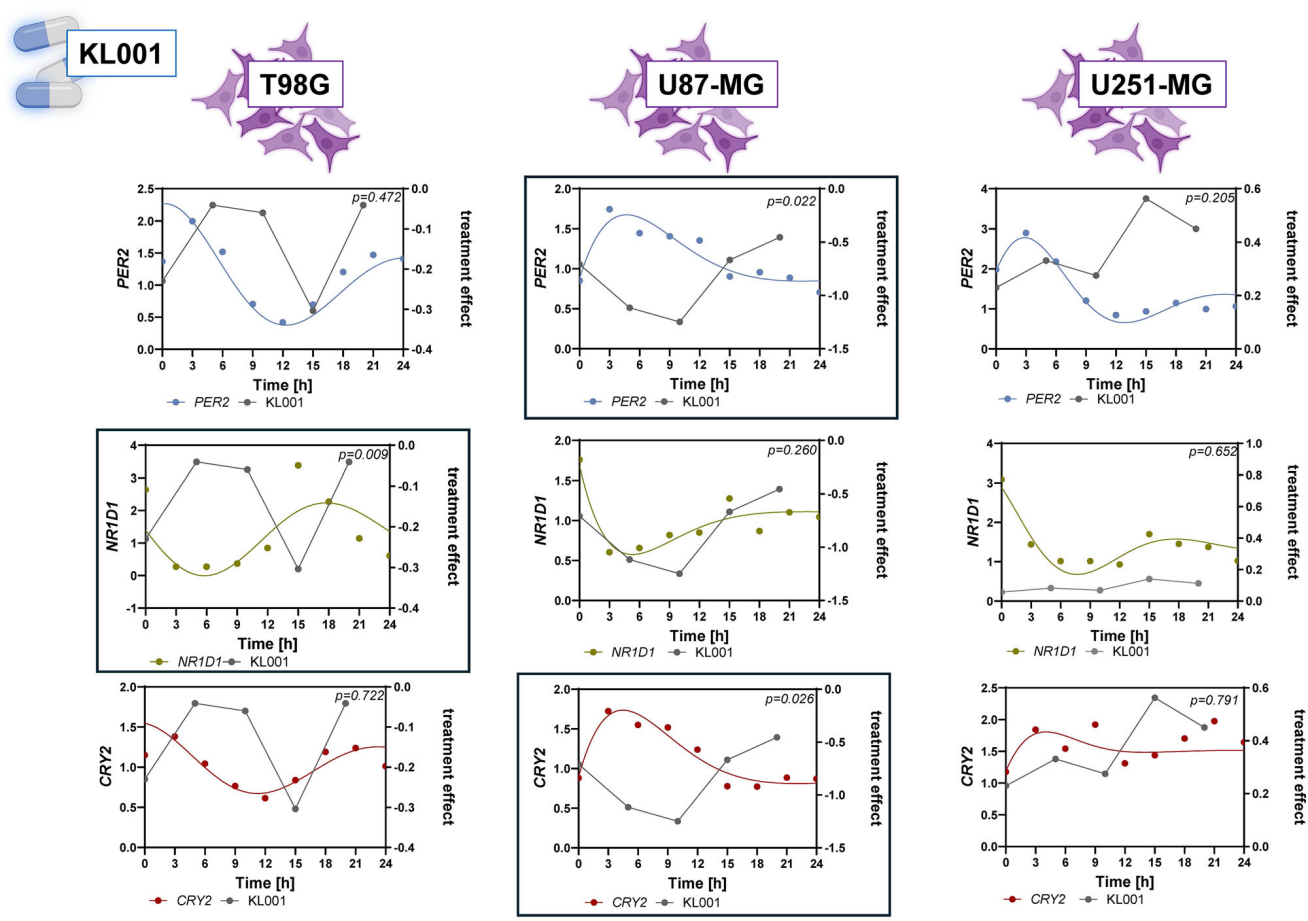
Gene expression is correlated with time-dependent treatment response profiles. Treatment effects were correlated with the gene expression data obtained by q-RT-PCR using Pearson correlation. The figure shows the scenarios displaying significant correlations (*p* < 0.05) in at least one cell line. Significant correlations are shown in boxes. All data were obtained from three independent biological replicates each containing three technical replicates. Icons were generated in Biorender.com.

Building on the observed time-dependent treatment effects, we next explored whether these responses were associated with the expression patterns of core circadian clock genes. To do so, we performed correlation analyses between the measured treatment effects and previously obtained circadian gene expression data (Fig. 3**;** Supplementary Figs. 5–7, Supplementary Table 2).

We found several significant correlations between KL001 sensitivity and components of the negative limb of the circadian clock. In T98G cells, KL001 treatment effect negatively correlated with *NR1D1* expression (*p* = 0.009, *r* = –0.96). In U87-MG, KL001 effect showed strong negative correlations with both *PER2* (*p* = 0.022, *r* = –0.93) and *CRY2* (*p* = 0.026, *r* = –0.922). These results suggest that KL001 efficacy may be modulated by the expression of genes involved in circadian repression.

Previous studies have reported a positive association between *BMAL1* expression and sensitivity to TMZ^8–10^. While we did not observe a statistically significant correlation between *BMAL1* and TMZ response in our data, we noted a moderately strong positive correlation in T98G cells (*p* = 0.116, *r* = 0.780), in line with earlier findings (Supplementary Fig. 5).

No further significant correlations were found regarding the other drugs used in this study (Supplementary Figs. 5–7, Supplementary Table 2).

**Fig. 4:**
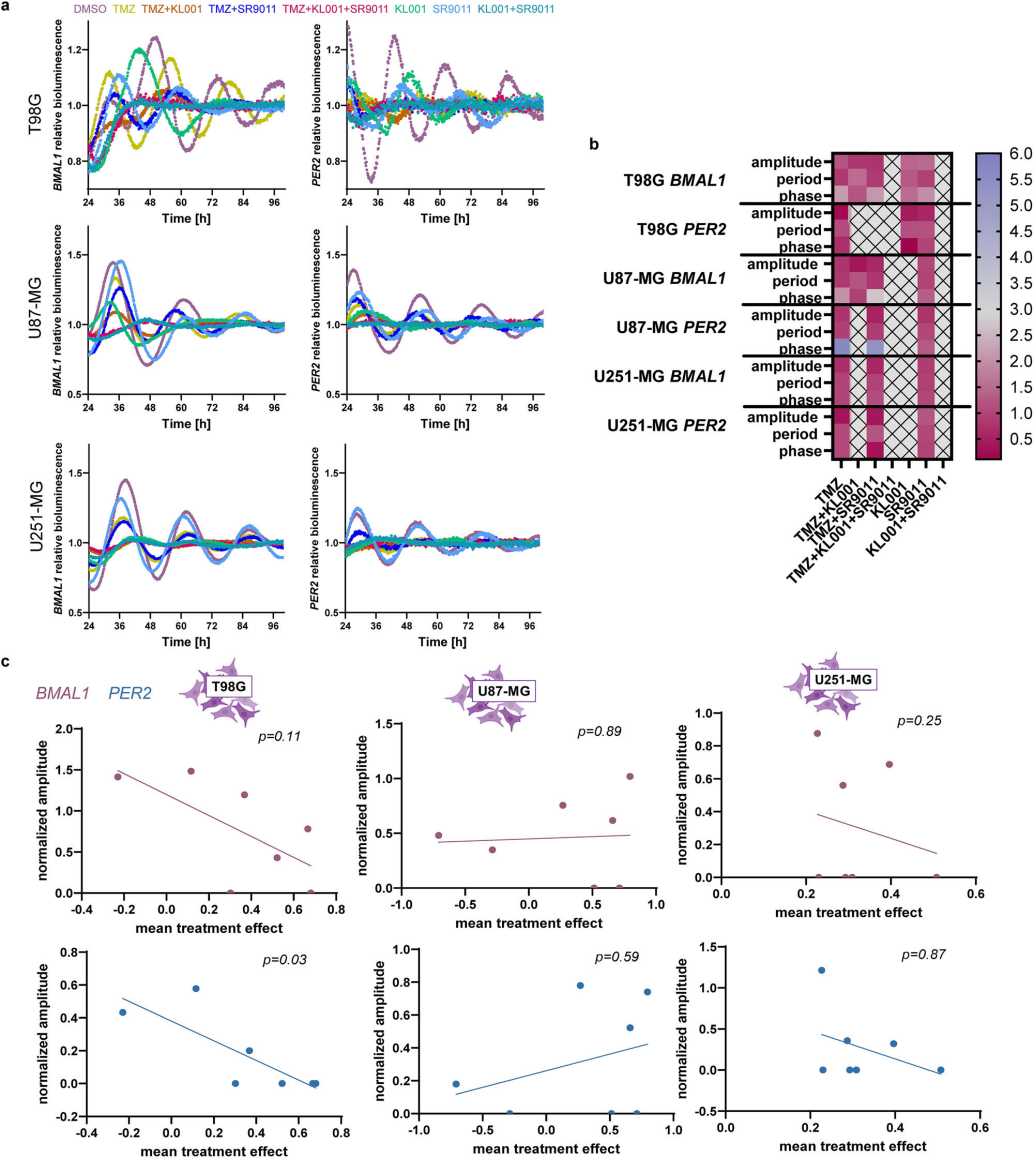
Treatment impacts the circadian profile which is correlated to mean treatment effect. **a** GBM cell lines were transduced with luciferase reporters for *PER2* and *BMAL1* promotor, synchronized and treated at 0 h after synchronization with the indicated treatments. Bioluminescence was recorded over 4 days. **b** The circadian parameters normalized to the DMSO control derived from the bioluminescence data. **c** The Pearson correlation between mean treatment effect and normalized amplitude under treatment. Bioluminescence data acquisition was performed at least twice with 3 replicates. Icons were generated in Biorender.com.

To evaluate whether treatment itself perturbed circadian rhythmicity, we treated cells transduced with *BMAL1* or *PER2* promoter-driven luciferase constructs using the same drug conditions as in the proliferation assays. Circadian bioluminescence profiles were recorded over a 4-day period (Fig. 4a, b).

Many treatments led to pronounced alterations in circadian rhythms, including a complete or partial loss of oscillatory behavior. We quantified the remaining circadian strength by calculating the amplitude relative to DMSO-treated controls. For partially arrhythmic conditions, amplitude was estimated from the mean-to-maximum luminescence values.

In T98G cells, we observed a significant negative correlation between *PER2* amplitude and mean treatment effect (*p* = 0.03, *r* = –0.802), suggesting that higher treatment efficacy was associated with greater disruption of *PER2*-driven rhythms. A similar, though not statistically significant, trend was observed for *BMAL1* (*p* = 0.11, *r* = –0.657). In contrast, no consistent correlations between amplitude and treatment effect were observed in U87-MG or U251-MG cells (Fig. 4c).

It has been reported that Luminescence reduction can be a result of reduced cell viability rather than alterations of biological processes^26^. To ensure that the correlation we found was not the result of reduced cell viability we assessed cell viability in parallel and did not find any significant correlation to Lumicycle amplitude (Supplementary Fig. 8). This suggests that the changes in amplitude are due to alterations in the circadian clock network rather than viability.

These findings imply that the extent to which treatment disrupts circadian amplitude may serve as a predictive marker for treatment response in certain GBM phenotypes. However, this predictive value appears to be cell line–specific and may depend on intrinsic clock robustness or additional, yet unidentified, genetic or molecular factors.

### Genetic disruption of core-clock genes alters drug sensitivity and circadian dynamics

To further probe the role of the circadian clock in treatment response, we generated CRISPR-Cas9 knockouts (KOs) of three key clock components—*BMAL1, PER2*, and *NR1D1*—in the T98G glioblastoma cell line. We selected T98G cells primarily due to its robust circadian rhythmicity, especially on the RNA level, which was not observed in the two other cell lines. We therefore hypothesized that KO of clock components would have more profound effects in a circadian healthy cell line like T98G, and thus a circadian healthy cell line would represent a good choice for the initial model development. The focus on a single cell line for genetic manipulation shows the validity of our approach, still it introduces limitations, and we aim to expand genetic clock manipulation to less circadian robust cell lines in future studies.

**Fig. 5:**
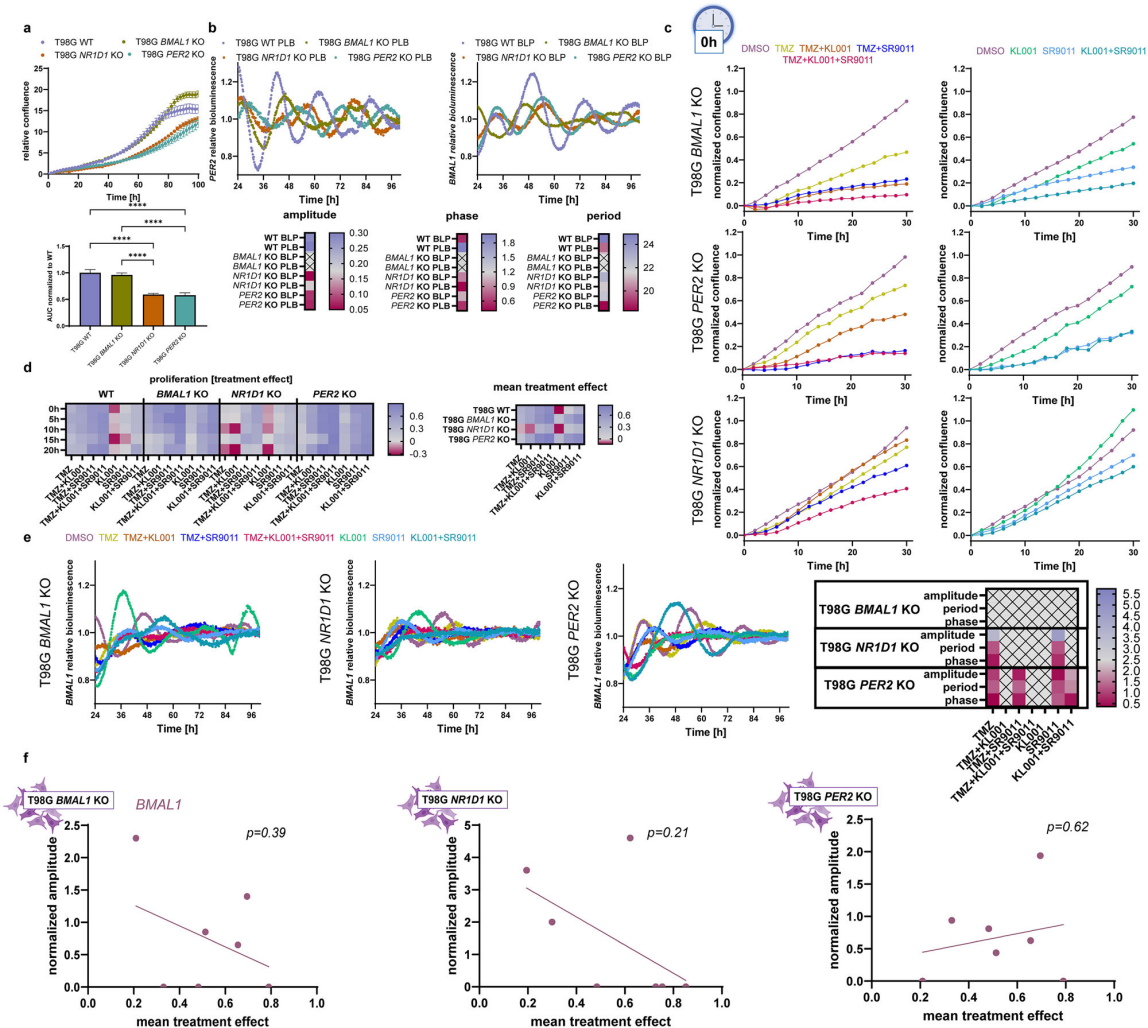
Genetic perturbation of the circadian clock influences chronotherapy treatment response. **a** KOs of core-clock proteins *BMAL1*, *PER2* and *NR1D1* were generated in T98G cells and proliferation was measured in untreated cells. **b** Circadian profiles of *PER2* and *BMAL1* bioluminescence reporters were recorded in T98G WT and clock KO cells and circadian parameters were calculated. **c** Proliferation curves for the treatment at 0 h after synchronization. **d** The heatmaps of treatment effects calculated as 1-AUC comparing T98G WT to the clock KOs. **e** The alterations of the circadian profiles under treatment at 0 h after synchronization for the clock KOs. **f** The Pearson correlation between amplitude under treatment normalized to DMSO derived from the bioluminescence recordings and mean treatment effects. Proliferation measurements were performed in three independent biological replicates with three technical replicates each and bioluminescence measurements were repeated at least twice with three replicates. Significance analysis was performed by one-way ANOVA, *p < 0.05, **p < 0.01, ***p < 0.001, ****p < 0.0001. Error bars represent standard deviation. Icons were generated in Biorender.com.

Loss of *NR1D1* and *PER2* significantly reduced proliferation relative to wild-type (WT) cells (*p* < 0.0001; Fig. 5a). All three KOs exhibited altered circadian profiles (Fig. 5b). Consistent with its central role, *BMAL1* KO led to arrhythmicity, while *PER2* and *NR1D1* KOs retained rhythmicity but with reduced amplitude and shifts in phase and period. These changes likely reflect partial compensation by paralogous genes.

Drug response analysis revealed that core-clock gene disruption significantly impacted treatment sensitivity in a time- and drug-dependent manner (Fig. 5c, d**;** Supplementary Figs. 9–11). Specifically: (1) TMZ Resistance: All three KO lines displayed increased resistance to TMZ, most notably in *NR1D1* KO cells, which were resistant across all timepoints (*p* = 0.004 vs WT). *PER2* KO also exhibited significantly reduced sensitivity (*p* = 0.004 vs WT), except at 15 h (*p* = 0.41 vs WT). In *BMAL1* KO cells, TMZ resistance was only significant at 15 h and 20 h (*p* = 0.02 for both vs WT). (2) KL001 Sensitivity: While KL001 had a negative or no effect in WT cells, both *BMAL1* and *PER2* KOs showed enhanced sensitivity across multiple timepoints. *BMAL1* KO displayed significant treatment effects at 0, 5, and 10 h (*p* = 0.02 vs DMSO). *PER2* KO cells responded significantly at all timepoints (*p* = 0.0001 vs DMSO). In contrast, *NR1D1* KO abolished the negative treatment effect observed in WT cells at 15 h but did not show a significantly improved mean response (*p* = 0.90 vs WT). (3) SR9011 Sensitivity: WT T98G cells did not respond to SR9011 alone. In contrast, both *BMAL1* and *PER2* KO cells exhibited strong and consistent sensitivity across all timepoints (*BMAL1* KO: *p* = 0.0001; *PER2* KO: *p* < 0.0001 vs DMSO). *NR1D1* KO cells showed increased sensitivity only at 15 h (*p* = 0.001 vs DMSO). (4) KL001 + SR9011 Combination: WT cells had a moderate response to the combination. This was markedly enhanced in *BMAL1* (*p* = 0.0003 vs WT) and *PER2* KO cells (*p* < 0.0001 vs WT), but not in *NR1D1* KO (*p* = 0.70 vs WT), where the effect was significant only at 15 h and reduced at 20 h (*p* = 0.031 vs WT).

To assess reproducibility, we generated *PER2* KO in U87-MG cells, which mirrored the T98G results (Supplementary Fig. 12). TMZ resistance was increased in U87-MG *PER2* KO, with significant reductions at 15 h (*p* = 0.004 vs DMSO, *p* < 0.0001 vs WT) and 20 h (*p* = 0.006, *p* = 0.0004). In line with T98G, *PER2* KO U87-MG cells showed significantly enhanced sensitivity to SR9011 (*p* = 0.01 vs WT) and KL001 + SR9011 (*p* = 0.003 vs WT).

### Circadian disruption in clock KOs alters temporal drug response

Clock gene knockouts disrupted circadian rhythms under treatment (Fig. 5e), with most conditions resulting in a loss of rhythmicity. Notably, the previously observed correlation between *BMAL1* amplitude and mean treatment effect in WT cells was weakened in the KO lines (Fig. 5f).

*PER2* KO cells exhibited a near-complete loss of time-dependency in treatment response, except for the TMZ + KL001 and TMZ + SR9011 conditions. In contrast, *BMAL1* and *NR1D1* KO cells retained time-dependency, though the optimal timepoints shifted. For example, SR9011 became time-independent in *BMAL1* KO cells, while KL001 + SR9011 was time-independent in *NR1D1* KO cells.

Given the observed correlation between *PER2* amplitude and treatment effect in WT cells—but not for *BMAL1*—these findings suggest that PER2 plays a particularly strong role in mediating chronotherapy responsiveness. Its loss most profoundly altered both treatment sensitivity and timing effects.

Altogether, these results support a strong and consistent relationship between circadian clock integrity and treatment efficacy in GBM cells. Genetic disruption of key clock components alters drug sensitivity, modifies circadian rhythms, and reshapes the temporal dynamics of treatment response. These effects were observed across multiple cell lines, underscoring the potential of circadian phenotyping as a predictive framework for optimizing personalized chronotherapy.

### A mechanistic PK-PD model recapitulates circadian variation in TMZ efficacy and predicts optimum treatment time points

**Fig. 6:**
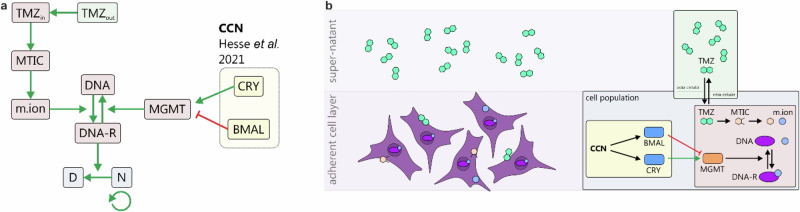
Model scheme of the employed TMZ PK-PD-cell population model. **a** Flow chart of the model components of the PK sub-section (green), the PD sub-section (red), and the cell population sub-section (blue). Extracellular TMZ (TMZ_out_) is internalized leading to accumulation of intracellular TMZ (TMZ_in_). Here, TMZ undergoes a series of activating reactions forming 3-methyl-(triazen-1-yl) imidazole-4-carboximide (MTIC) and subsequently the methyl diazonium ion (m.ion). This last species induces alkylation of DNA forming alkylated DNA adducts (DNA-R). This reaction in turn is reversed by O6-methylguanine DNA methyltransferase (MGMT). The DNA-R adduct induces a cytotoxic effect on the cell population (N) leading to accumulation of dead cells (D). For the core-clock machinery (yellow), only the components establishing the linkage to the TMZ PK-PD-cell population model are shown. While MGMT positively correlates with CRY expression, the relation to BMAL was described to be anti-phasic. For the detailed set-up of the CCN model sub-part the reader is referred to previously published work. **b** Scheme illustrating how the described model aligns with the in vitro experimental set up in cell culture experiments.

To explore the mechanistic relationship between the circadian clock and time-dependent efficacy of TMZ, we developed an integrated pharmacokinetic-pharmacodynamic (PK-PD) model that couples TMZ activity to the core-clock network (Fig. 6). This model simulates both (A) proliferation-time curves and (B) circadian fluctuations in the area under the curve (AUC) of cell proliferation, thereby enabling prediction of time-of-day–dependent treatment responses. These time response curves allowed us to identify optimum time points for treatment.

Our approach is the first to implement a detailed, biologically validated model of the core-clock machinery in the form of a coupled ODE system previously published by our group^18^. This ODE system is connected to a TMZ efficacy model consisting of three sub-components: (1) a pharmacokinetic (PK) component describing cellular uptake of TMZ, (2) a pharmacodynamic (PD) component describing molecular activation of and DNA alkylation induced by TMZ, and (3) a cell population model describing how alkylated DNA affects cellular proliferation. The efficacy model (i.e., PK-PD-cell population) is linked to the core-clock machinery (i.e., CCN) via the detoxifying enzyme O6-methylguanine DNA methyltransferase (MGMT) reversing TMZ induced DNA alkylation. This MGMT enzyme in turn has been described to be co-expressed with CRY2 and to follow *BMAL1*^27^ expression in an anti-phasic manner, offering two connection points to the core-clock machinery.

### Model performance in wild-type cells and core-clock knockouts

**Fig. 7:**
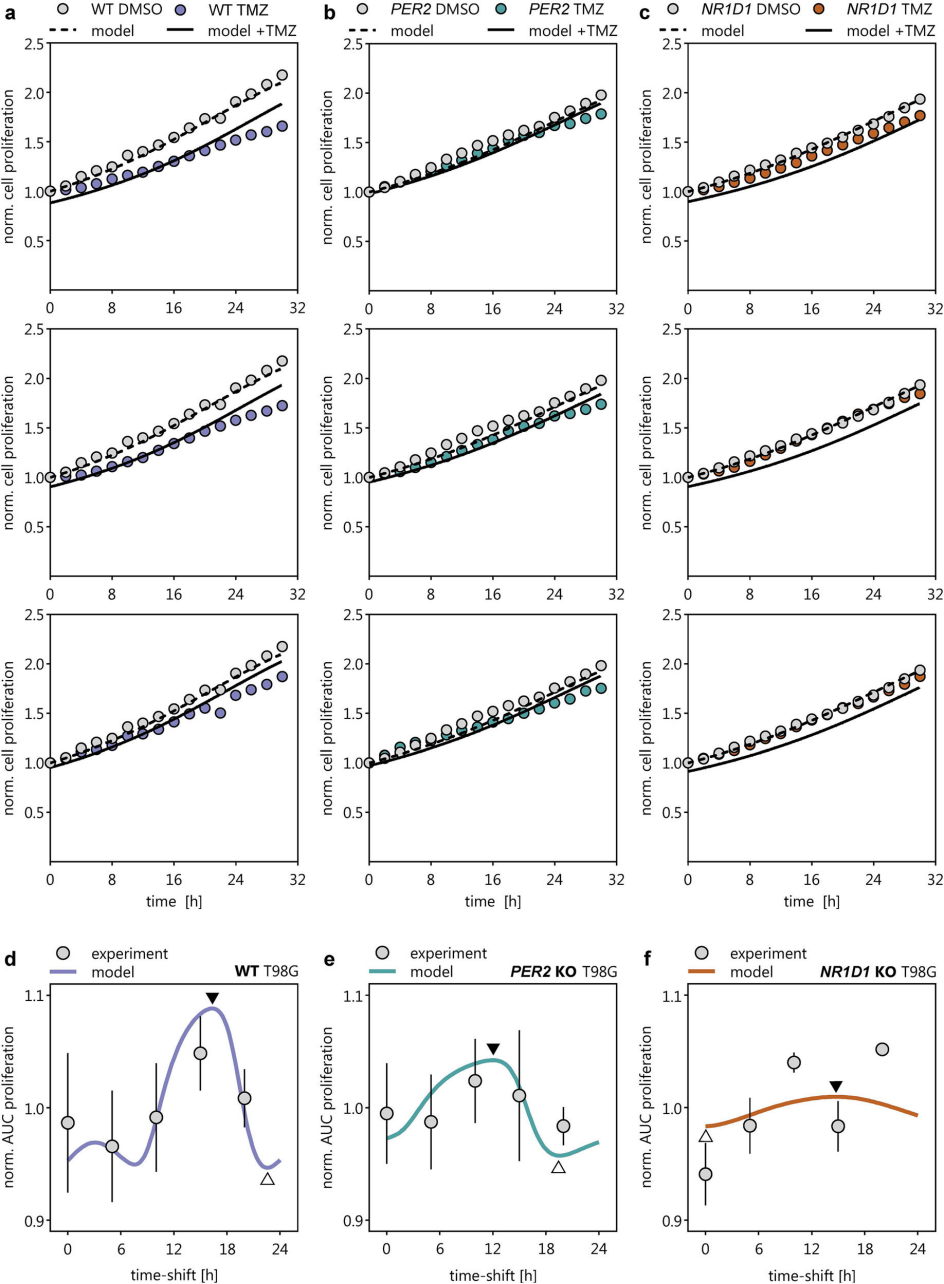
Simulation outcomes of coupled core-clock machinery-TMZ efficacy model. Cell population proliferation curves obtained from experimental studies (symbols) and simulations (lines) for **a** WT-, **b** *PER2* KO-, and **c** *NR1D1* KO T98G cell line. **d**–**f** 24 h fluctuation curves of TMZ efficacy expressed as area under the proliferation curve (AUC) for **a** WT-, **b** *PER2* KO-, and **c** *NR1D1* KO T98G cell line (error bars represent standard error of mean). Curve maximum (white triangle) and minimum (black triangle) are indicated to mark the model found optimum time point for treatment. This optimum time point corresponds to the curve minimum as proliferation inhibition is highest at this point.

We first simulated TMZ efficacy in wild-type T98G cells, which retain an intact core-clock. The model captured key experimental features, including the amplitude and periodicity of circadian fluctuations in TMZ response. Simulated proliferation-time profiles and AUC values closely matched experimental observations (Fig. 7a, d), supporting the validity of the model under baseline conditions.

**Fig. 8:**
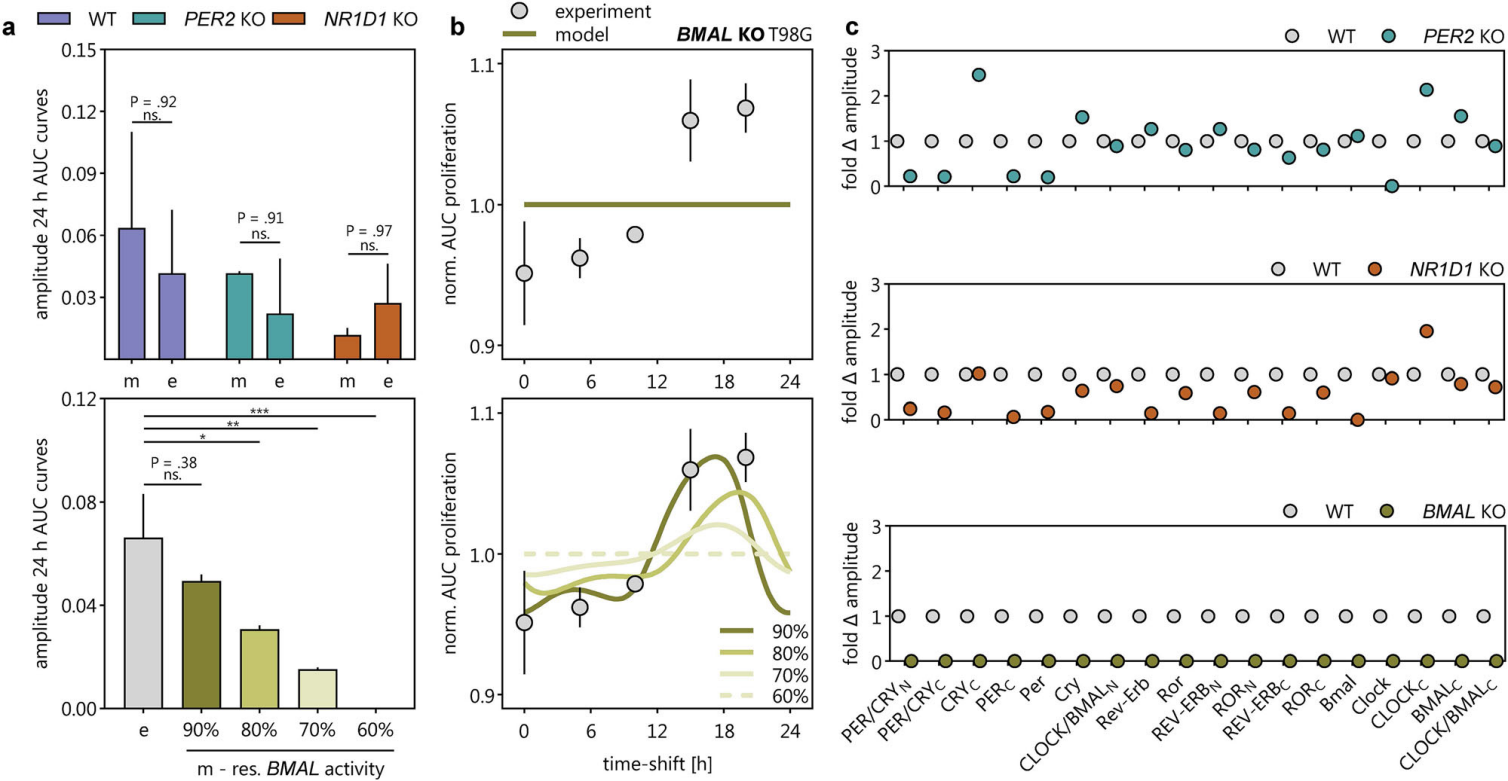
Alignment of coupled core-clock machinery-TMZ efficacy model outcome with experimental observations. **a** Amplitude of 24 h fluctuations of AUC proliferation was compared between experimental (e) observations and model (m) outcome (error bars represent standard error of mean). For WT, PER2, and NR1D1, we conducted a two-way ANOVA test using Šídák’s multiple comparisons test (*α* = 0.05; ns. not significant). For BMAL, we conducted a one-way ANOVA test using Dunnett multiple comparison correction (*α* = 0.05; *P < 0.05, **P < 0.005, ***P < 0.0005). **b** Model outcomes for *BMAL* knock-out simulations using varying residual activities for BMAL (10% in top plot as used for all other knock-out simulations; 60, 70, 80, and 90% in bottom plot to account for partial knock-out of *BMAL*). **c** Effect of tested knock-outs on all components of the core-clock machinery (results for 10% residual activity).

We next tested whether the model could replicate TMZ efficacy profiles in cells with genetic disruption of core-clock genes. (1) *PER2* KO: The model accurately predicted the overall treatment effect and circadian dynamics observed experimentally in *PER2* KO cells. Both the magnitude of proliferation inhibition and the shape of the 24-h fluctuation curve aligned closely with empirical data (Fig. 7b, e). (2) *NR1D1* KO: Simulations slightly overestimated TMZ sensitivity and underestimated the amplitude of circadian fluctuations. Despite these minor discrepancies, the differences between modeled and experimental outcomes were not statistically significant (Fig. 7c, f**;** Fig. 8a). (3) *BMAL1* KO: Notably, simulations predicted a complete loss of circadian modulation in TMZ efficacy due to full collapse of oscillations in the core-clock network (Fig. 8b), as expected from the central role of *BMAL1*. However, experimental data still showed residual circadian fluctuations in TMZ efficacy with relevant amplitude (Fig. 8c), indicating a mismatch between model prediction and biological outcome.

To address this discrepancy, we performed additional simulations assuming partial, rather than complete, *BMAL1* knockdown. Restoring as little as 30% of *BMAL1* activity was insufficient to recapitulate observed rhythmicity. At least ~70% residual *BMAL1* activity was required to reintroduce time-of-day–dependent fluctuations in treatment response (Fig. 8b), suggesting the presence of compensatory mechanisms maintaining residual circadian function.

With our model we lay the foundation for a tool to generate patient individual TMZ chronotherapeutic recommendations based on gene expression data.

## Discussion

Our findings support the hypothesis that circadian timing modulates glioblastoma response to chemotherapy via mechanisms tied to both drug metabolism and intrinsic clock-regulated pathways. Using three GBM cell lines displaying different circadian profiles we found a significant time-dependency in the majority of conditions. Importantly, both the drug effect and its temporal dynamics differed between clock phenotypes.

Clock modulation, either by pharmacological or genetic means further altered treatment response. This suggests that such modulation could enhance the therapeutic efficacy of TMZ when used in combination. Interestingly, our results also indicate that TMZ treatment itself can modify the circadian profile, aligning with prior studies showing bidirectional interactions between the clock and chemotherapy agents^25,28^. In T98G cells, we found a significant negative correlation between *PER2* amplitude and mean treatment effect, implying that treatment-induced alterations in circadian profiles may serve as predictors of treatment efficacy. However, such a correlation could not be observed in the other two cell lines. This suggests the need for the development of more complex models and highlights the need for personalization in larger experimental cohorts in future studies.

Notably, our results support previous studies demonstrating the potential of pharmacological clock modulation as a complement or even an alternative to standard TMZ therapy^20,21,25,28–34^. For instance, in U87-MG cells, SR9011 consistently exhibited equal or greater efficacy than TMZ across all timepoints. Prior studies have also reported that clock modulators exert stronger effects in GBM cells than in healthy cells, highlighting their therapeutic potential^20,29^. However, our findings do not suggest a uniform benefit of clock modulation: for example, KL001 exerted negative effects in both T98G and U87-MG cell lines, indicating that the effect of clock-targeting agents is highly cell line-dependent. We found profound resistance of U87-MG cells to KL001, which is surprising given U87-MG’s stem-like nature^35^ and the reported high sensitivity of glioblastoma stem cells (GSCs) to clock modulators relative to healthy cells and differentiated GBM cells^29^. However, multiple studies reported the reduction of stemness when U87-MG were cultured as attached monolayers in serum-containing medium as conducted in our study compared to tumorsphere cultures^36–38^. We believe that the more differentiated nature of U87-MG grown under these conditions contributed to the observed higher resistance to KL001 seen in our data.

Using genetic perturbation of core-clock genes (*BMAL1*, *PER2*, *NR1D1*), we found that disruption of circadian function led to altered treatment responses. The KOs of core-clock elements generally showed reduced sensitivity to TMZ and increased sensitivity to clock modulators, consistent with the idea that a functional clock enhances TMZ efficacy.

We chose to treat the cells at predetermined IC_50_ drug concentrations to standardize our protocol across the cell lines used. Treatment at or near IC_50_ concentrations has been conducted previously in similar studies^39,40^. However, we cannot exclude that treatment at such a relatively high dose might have led to the selection of resistant clones^41^. We selected a short treatment window and monitored cell proliferation from the beginning on to reduce such effects. Although our experimental results showed that proliferation curves began to separate as early as 2 h after treatment start, recent single-cell transcriptome studies suggest that cell populations drift rapidly following drug exposure^42,43^. The treatment at only one drug concentration therefore presents a limitation of the study and should be addressed in the future by incorporating treatment at lower dosage as well as dose-gradient protocols.

**Fig. 9:**
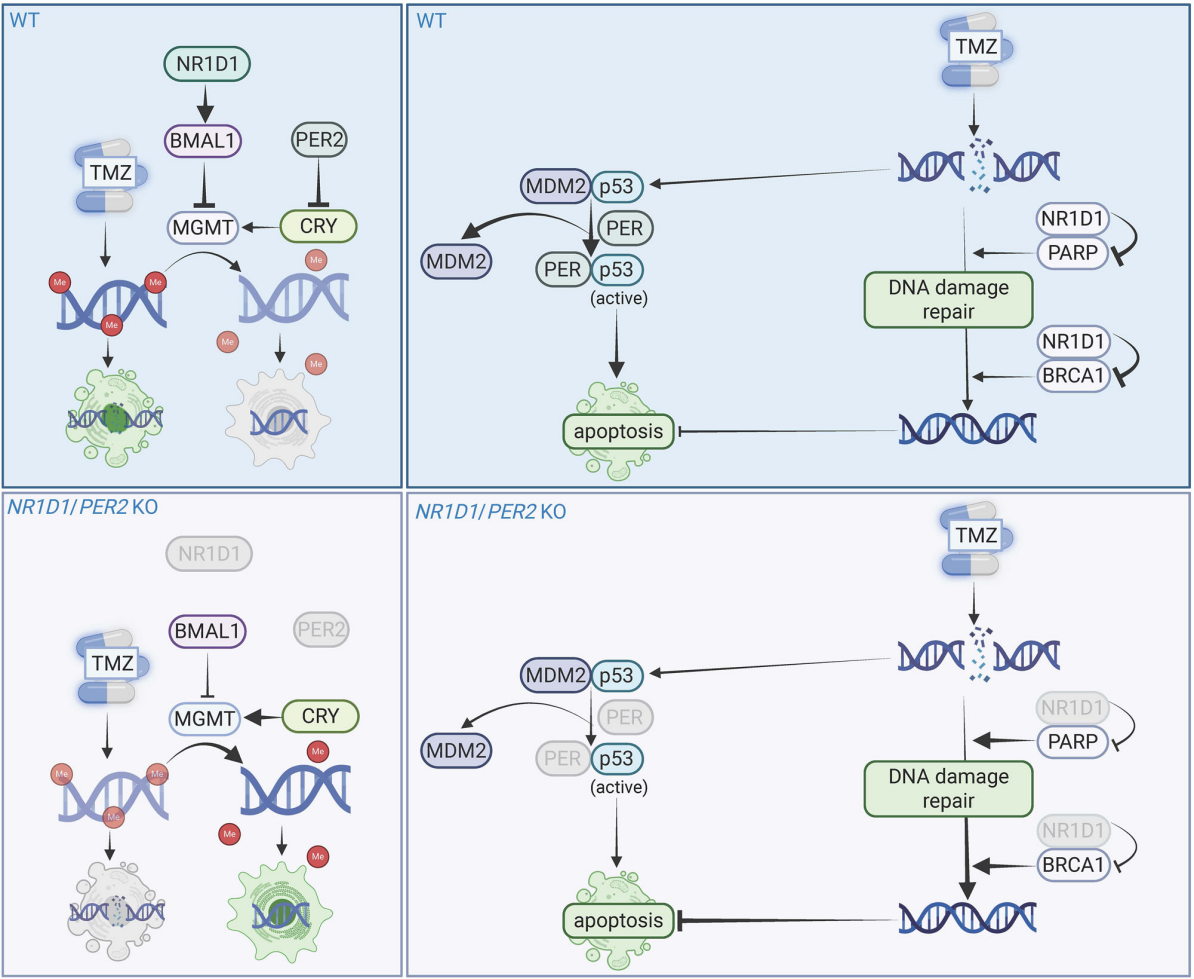
Model on the impact of clock KOs on TMZ sensitivity. **Left panel:** TMZ sensitivity was shown to be coinciding with the peak of BMAL1 and BMAL1 and MGMT were shown to stand in an antiphase relationship to each other. MGMT is the main detoxifying enzyme of TMZ and implicating in removing TMZ-mediated methyl groups from DNA thus preventing DNA damage and apoptosis. The model predicts an abolished inhibitory effect of PER2 in the PER2 KO cells, therefore promoting the activation of MGMT by CRY. Additionally, the NR1D1 KO predicts lower BMAL1-dependent inhibition of MGMT. **Right panel:** PER2 was shown to compete with MDM2 for the binding of p53 and to activate p53 in this process. p53 is induced by DNA damage and induces apoptosis. In the PER2 KO p53 activation following TMZ-mediated DNA damage is expected to be reduced explaining the higher resistance to TMZ. NR1D1 was shown to inhibit DNA damage repair by PARP and BRCA1. In the NR1D1 KO cells DNA damage repair after TMZ exposure is expected to be enhanced contributing to enhanced resistance to TMZ. The figure was created in Biorender.com.

Our mathematical model was able to recapitulate experimental results, i.e., the higher observed resistance to TMZ in the clock KOs, with a high predictive power, suggesting a link between individual clock profiles and personalized chronotherapy. This aligns with previous reports linking TMZ sensitivity to circadian timing, particularly the peak of *BMAL1* expression coinciding with the trough of *MGMT* and increased DNA damage-induced apoptosis^8–10^. In our KO cells, we observed a lower amplitude of *BMAL1* expression, which may result in elevated MGMT levels, thereby contributing to TMZ resistance. This BMAL1:MGMT regulatory axis is also reflected in our model predictions (Fig. 9).

Interestingly, the *BMAL1* KO effect was less pronounced than that of *PER2* or *NR1D1* KO. Mechanistically, TMZ results in apoptosis through DNA damage^22^, and a recent PK–PD model by Corridore et al. demonstrated circadian oscillations in double-strand breaks (DSBs) under TMZ, linked to cell cycle regulation and cyclic activation of the ATR/CHK1/p53 axis^15^. Given the tight interconnection between the cell cycle and circadian clock^44^, our data suggest additional mechanistic pathways: PER2, for instance, promotes p53 activation by competing with MDM2, and thus *PER2 KO* may reduce p53 signaling and promote resistance as observed for TMZ treatment^45^. Similarly, NR1D1 has been shown to form inhibitory complexes with DNA repair proteins PARP1 and BRCA1^46–48^, suggesting that its loss could enhance DNA repair capacity and reduce TMZ toxicity. These mechanisms likely explain the higher resistance observed in *PER2* and *NR1D1* KO cells, compared to *BMAL1* KO. (Fig. 9).

The Corridore *et al*. mathematical model further identified DNA damage response modules (p53, ATR phosphorylation) as predictive of TMZ response and showed that combining TMZ with PARP or HR inhibitors (e.g., niraparib, RI-117) significantly improved treatment outcomes^15^. This aligns well with our data and supports the central role of DNA repair pathways in TMZ resistance. Through our simulation approach—coupling a TMZ PK–PD and cell population model with a core-clock network model—we accurately predicted the 24-h fluctuations in TMZ efficacy across different genetic backgrounds. Beyond prediction, the model also offered mechanistic insights: for example, *PER2* KO simulations showed upregulation of *CRY*, leading to increased MGMT expression and enhanced DNA repair. In contrast, *NR1D1* KO simulations showed *BMAL* downregulation, also contributing to reduced TMZ toxicity via derepression of MGMT (Fig. 9). Thus, our model may serve as a future tool to develop patient-specific chronotherapeutic regimens, adjusting dosing based on individual clock gene expression profiles for later applications in TMZ therapy. Future work will combine further development of pharmacokinetic models within the current model to take into account the actual distribution of TMZ across the body and thus derive optimal times of administration for patients in real clinical and treatment scenarios.

Moreover, our data show that genetic disruption of the clock reduces time dependence of treatment response. Especially, in *PER2* KO cells, most treatments became time-independent. The correlation between treatment efficacy and circadian amplitude also weakened compared to WT cells, suggesting a diminished influence of circadian rhythms. However, some time-dependent effects persisted, likely due to incomplete abolition of circadian oscillations in the KO models. Interestingly, we observed shifts in the timing of treatment sensitivity between WT and KO cells, indicating that partial disruption of clock function may differentially affect drug response timing depending on the agent used. Future studies should explore complete genetic or pharmacological ablation of the circadian system to clarify the extent to which time-dependency is causally linked to the clock.

While we were able to explain the majority of our experimental findings, some aspects are to be explored in the future. One intriguing finding is certainly that *BMAL1* KO and *PER2* KO displayed similar drug responses. This seems rather unexpected given that BMAL1 represents the main component of the positive arm of the circadian clock and PER2 represents the main component of the negative arm^49^. We believe that not yet known compensatory mechanisms, non-canonical feedback loops or non-clock related effects may be involved. Furthermore, due to the central role of BMAL1, a complete loss of rhythmicity both with regards to the Lumicycle data and the treatment response would be expected, which was also predicted by our model. However, the experimental data suggest some residual rhythmicity. A possible explanation could be the presence of non-canonical rhythms in the absence of BMAL1 or BMAL1 transcriptional activity. Non-canonical transcriptional loops, as well as posttranslational modification and redox oscillations have been proposed previously to function in the absence of a functional circadian clock, in line with our observations^50–54^. Intriguingly, Ray et al. reported retained rhythmicity in *Bmal1* KO mice, attributed to ETS transcription factor dynamics and redox cycles, even in the absence of light and temperature cycles^54^. However, mice were still given food, which could have been a confounding factor^54^. Furthermore, the technical validity of the obtained results have been questioned^55,56^ and the majority of studies point to loss of circadian rhythmicity in the absence of *Bmal1*^57^.

Of note, cells were synchronized before the measurements, which may have provoked temporal clock-independent oscillations. This would fit with the fact that we found rhythmic oscillations in our *BMAL1* KO cells only in the first hours after synchronization after which cells clearly lost rhythmicity. Another not yet fully explainable finding is the same treatment profile of T98G and U87-MG *PER2* KO cells regarding KL001 + SR9011 whereas the WT cells showed considerably different temporal treatment profiles. In particular, U87-MG WT cells showed higher proliferation compared to the DMSO control at 5, 10 and 15 h whereas proliferation was consistently reduced in T98G WT over all timepoints. In contrast, both KOs displayed enhanced sensitivity compared to WT over all timepoints. Given the different circadian profile and circadian treatment response in the WT cells, we would have expected that KO of the same gene would result in cell-line specific temporal treatment profiles, which was not the case here. We suggest that non-clock related effects related to the generally reduced clock function in the KOs, direct involvement of PER2 in drug PD or the presence of redundant pathways which couple to drug response in a similar manner in both cell lines may be at play here. Future work should therefore focus on expanding the current mathematical model based on the generated hypothesis-generating results and on applying appropriate experimental techniques to better understand such new observations.

While our model successfully recapitulates key aspects of the experimental data, we acknowledge that it does not yet capture the full complexity of glioblastoma. To further expand and refine our approach, the Endogenous Network Theory proposed by Ao et al. offers a promising framework for characterizing the dynamics of gene regulatory networks in GBM^58^. Notably, Yao et al. applied this theory to map the landscape of glioma and identified two stable and several transitional states, potentially relevant to GBM heterogeneity and recurrence^59^. Building on this, their model was also leveraged to optimize targeted combination therapies in GBM^60^. Incorporating such a systems-level perspective may enhance the explanatory power of future models and provide a more comprehensive understanding of treatment response. Furthermore, this approach could be instrumental in modeling temporal dynamics and treatment effects beyond TMZ, aligning with the broader goal of improving personalized therapeutic strategies in GBM.

Another limitation of the study is that MGMT methylation status and/or expression was not directly assessed across the circadian cycle or under treatment, and further work is needed to assure that the antiphase relationship between *BMAL1* and *MGMT* is a general principle. At the current point the relationship between *BMAL1* and TMZ resistance has been shown both in cellular models and in clinical datasets^8–10,61^. Moreover, a recent study could show a positive correlation between *NR1D1* and *MGMT* in a clinical cohort^61^. The same study also compared U87-MG cells showing low MGMT expression with U87-MG TMZ resistant cells showing high MGMT expression and found a positive correlation of MGMT with NR1D1 and a negative correlation with BMAL1^61^. These findings point to a generalizability of the relationship between BMAL1 and MGMT expression although not fully proven yet.

In line with this, direct combinatorial treatment of GBM cells with TMZ and an MGMT inhibitor instead of searching for the right treatment time window as in our study appears to be a viable option. However, a phase II clinical trial of TMZ and the MGMT inhibitor O6-BG displayed increased toxicity and did not yield any significant clinical benefit compared to TMZ alone^62^. Further clinical studies aiming to target MGMT in other cancers showed mixed results as recently reviewed^63^. Moreover, a computational study exploring this option judged it as unrealistic as their calculations predicted a nearly 100% MGMT inhibition would be needed to significantly lower the TMZ IC_50_ value in resistant cells^15^. While MGMT is certainly the main determinant of TMZ resistance, some studies suggest that alternative pathways such as AKT, β-catenin, and alternative DHC2-mediated DNA repair exist to confer TMZ resistance in MGMT negative cells^64,65^. We therefore think that as of now optimizing the timepoint of TMZ delivery seems like the more practical solution as it is applicable independent of MGMT status and does not require clinical approval of MGMT inhibitors.

Despite significant progress in understanding GBM biology and treatment, key challenges remain. These include elucidating the mechanistic basis of circadian modulation of drug response in GBM, determining the optimal timing of TMZ administration in clinical settings, and conducting large, prospective trials to validate chronotherapy strategies. Additionally, there is a need to better understand how circadian gene regulation influences treatment resistance and interactions within the tumor microenvironment (TME).

One of the major challenges in GBM treatment certainly is its high level of heterogeneity both regarding the tumor itself and the TME^66^. We utilized cell lines with different circadian profiles to capture heterogeneity, but admittedly our current in vitro model does not suffice in capturing the full complexity of GBM in patients. Future studies should therefore expand on the knowledge gained in our study to better recapitulate intra-tumoral heterogeneity. A tumorsphere cell model by Guldbergsson et al. recapitulated intra-tumor heterogeneity both in vitro and after transplantation into mice and a similar approach may represent a first step in expanding on the current analysis^67^. The main prospective goal however will be to transfer the knowledge gained from in vitro systems into clinical applications. This will be particularly challenging in the case of GBM due to its exceptionally high heterogeneity and plasticity in comparison to other tumors^66^. We therefore propose a combination of (1) developing a better understanding of how circadian chronotype and chronotherapy response correlate to biomarkers that are clinically accessible in appropriate in vitro models such as cell lines and primary cells and (2) appropriate clinical diagnosis capturing the intratumoral heterogeneity followed by prediction of the optimal chronotherapy based on the data gained in step (1). Several diagnostic methods are available for this purpose: First, studies using precision technology such as 3D neuronavigation during biopsy collection enable a comprehensive mapping of the tumor and underline the need to take multiple biopsies for an accurate representation of the tumor landscape^68–70^. Radiomics paired with machine learning represents an exciting non-invasive technique to map GBM^71^. This has been applied to capture the heterogeneity in *EGFRvIII* status^72,73^, *IDT* mutational status^74–78^, 1p/19q co-deletion^75^, *MGMT* status^75^, GBM subtype^79^ and tumor molecular pathways^80–83^. We developed a non-invasive protocol to profile clock phenotypes in healthy and ovarian cancer populations based on saliva sampling and could show a connection of clock phenotype with clinical drug response^16,84^. Such an approach, although warranting future comprehensive validation in GBM, may be convenient to acquire an estimate of the GBM clock in patients especially in the light of recent evidence that GBM synchronized to the host’s central circadian clock^85^. Liquid biopsy represents another non-invasive approach to directly monitor the tumor over time-series and can be helpful to assess tumor heterogeneity over multiple timepoints^86^. Such an approach may be used as a complement to the saliva-based method to assess the tumor circadian rhythm. Furthermore, deep learning and scRNA-Seq has enabled the prediction of drug responses from cell line data to single cells in patient tumors^43,87–90^. Recently, Wang *et al*. utilized TCGA and CGGA expression data from 1042 glioma samples, and were able to identify CCG (Clock Core Gene) patterns associated to distinct tumor behavioral states as a novel molecular marker to define the GBM chronotype^91^. Additionally, they could show that single cells from the same tumor differed in their CCG patterns^91^. This finding was supported by De La Cruz Minyety *et al*. which found that anatomical subregions of the same GBM tumor displayed distinct clock profiles^92^. It is therefore expected that they would also potentially benefit from different treatment time windows.

Such approaches will certainly prove highly useful to transfer our cell line data to GBM subpopulations and optimize GBM chronotherapy. Although methods such as focused ultrasound-activated nanoparticles enable the targeted release of drugs into specific brain regions^93^, TMZ is administered systemically and a more applicable approach would therefore be to focus on optimizing chronotherapy for the most aggressive subpopulations.

While our current study uses isolated tumor cells, but it has to be noted that GBM does not exist in isolation but in a tight interplay with the TME^94^. Thus our experimental and mathematical models offer a system to study GBM circadian behavior and reaction to treatment timing, which despite its simplification allow for a mechanistic analysis and share valuable insights into possible chronotherapy applications. GBM is known for its highly immunosuppressive microenvironment, which was shown to be enforced by clock-dependent reprogramming of Tumor Associated Macrophages (Microglia, Bone Marrow Derived Macrophages) and Myeloid Derived Suppressor Cells^95^. Additionally, a tight crosstalk between GBM and brain cells (neurons, astrocytes and oligodendrocytes) has been demonstrated^94^. Although the influence of the TME on the GBM clock has not been extensively researched yet, current evidence suggests that the TME does have an impact on the GBM clock: Li *et al*. demonstrated that tumor-promoting microglia secreted *miR-7239-3p* containing exosomes which subsequently reprogrammed the GBM molecular clock^96^. Furthermore, circadian clock gene profiles differed between the tumor periphery (high immune infiltration) and tumor core (low immune infiltration) in the IvyGap GBM dataset^92^. A study by our group showed that benign and tumor-associated fibroblasts differently impacted the clock of colorectal cancer cells, which further underlines that the TME can impact the tumor circadian profile^97^. Tumor associated reactive astrocytes (TARAs) and tumor associated oligodendrocytes have not yet been shown to influence the GBM clock, but it is known that they alter GBM biology by, for instance the secretion of soluble factors or transfer of mitochondria^94^. Therefore, it is not unlikely that they would impact the circadian clock as well. Intriguingly, Venkataramani et al. identified a direct signaling from glutamatergic neurons to GBM cells via AMPA receptors suggesting that GBM is influenced by neuronal networks^98^. In line with this, a recent study by Gonzalez-Aponte et al. showed that GBM synchronized to the host’s central clock via glucocorticoids^85^. Using mouse models, the authors showed that circadian rhythms were lost in *Vip* (Vasoactive Intestinal Peptide) and *Gr* (Glucocorticoid Receptor) KO mice^85^. Given the high heterogeneity of the GBM TME^66^, expanding our current experimental and mathematical framework to depict various states of the TME, i.e., using appropriate co-culture models would be extremely interesting for prospective clinical transfer.

Given the critical role of the tumor-associated immune microenvironment in GBM progression and treatment resistance, immunotherapy has been explored as a potential alternative to current standard-of-care protocols. However, these approaches have shown limited success in GBM to date^99^. Recent findings in other cancer types suggest that incorporating circadian timing into immunotherapy—immune-chronotherapy—can enhance therapeutic efficacy^100^. This is consistent with the well-established role of the circadian clock in regulating immune cell function and anti-tumor immune responses^101^. Additionally, the efficacy of CAR-T cell therapies has been shown to improve when combined with RORγ agonizts in several solid tumor models^102,103^. Importantly, there is compelling evidence that the circadian clock in GBM cells influences the function and composition of GBM-associated immune cells^95^, highlighting a promising opportunity for applying immune-chronotherapy strategies in GBM. Future work integrating circadian biology into immunotherapeutic approaches may help overcome current limitations and offer a new avenue to enhance treatment response in this challenging disease.

Our study represents an initial step toward addressing these challenges. By integrating experimental approaches with mechanistic mathematical modeling, we provide a framework for exploring the impact of the circadian clock on TMZ response. This combined strategy has the potential to guide the development of individualized chronotherapy protocols, tailored to the circadian characteristics of each patient. Such an approach may ultimately contribute to more effective and personalized GBM treatment strategies.

In summary, our data establish a strong link between circadian phenotypes and TMZ pharmacodynamics in GBM, supported by both experimental results and mechanistic modeling. Circadian regulation—particularly of DNA damage repair pathways—appears to significantly influence treatment efficacy. Moreover, chronopharmacological modeling emerges as a promising tool to predict optimal treatment windows, ultimately enabling more effective and individualized GBM therapies.

Future research should focus on translating these insights into clinical settings by designing personalized chronotherapy trials in GBM patients, with the goal of improving therapeutic outcomes through circadian-informed treatment strategies.

## Methods

### Cell lines and genetic perturbations

The human glioblastoma cell line T98G, originally derived from a primary tumor of a 61-year-old Caucasian male, and widely used in GBM research was used as an in vitro cellular model. T98G cells harbor deletions in *CDKN2A*, *PART3*, *TP53*, and *PTEN*, and are *IDH* wild-type (*IDH*-WT). For assessment of phenotypic variation in drug response, U87-MG cells, derived from a Caucasian male of unknown age, which exhibit mutations in *NF1*, *PTEN*, *TP53*, and *TERT* (*IDH*-WT), and U251-MG cells, derived from a 75-year-old Caucasian male, with mutations in *PTEN*, *TERT*, and *TP53* (*IDH*-WT), have been included. The MGMT expression and promotor status was reported as follows: T98G *MGMT* promotor methylation negative, high MGMT expression^64^; U87-MG *MGMT* promotor methylation positive, low MGMT expression^64,104^; U251-MG *MGMT* promotor methylation positive, low MGMT expression^105^.

All cell lines were authenticated by the Eurofins Cell Line Authentication Service. T98G and U87-MG cells were cultured in Minimum Essential Medium (MEM; Gibco, Thermo Fisher Scientific, Waltham, MA, USA) supplemented with 2 mM GlutaMAX™, 1 mM sodium pyruvate, 0.1 mM non-essential amino acids (NEAA), 1% penicillin-streptomycin, and 10% fetal calf serum (FCS). U251-MG cells were cultured in RPMI 1640 medium (Gibco) supplemented with 2 mM GlutaMAX™, 1% penicillin-streptomycin, and 10% FCS. CRISPR-Cas9-mediated knockouts of key core-clock genes (*BMAL1*, *NR1D1*, and *PER2*) were generated as previously described^106^.

### Circadian phenotyping

Circadian rhythms in cell lines were characterized using the bioluminescence reporters *PER2::LUC-Blasticidine* (PLB) and *BMAL1::LUC-Puromycin* (BLP) encoding for the *BMAL1* or *PER2* promotor fused to luciferase as described previously^106–108^. Briefly, lentiviral particles were produced in HEK-293 cells and glioblastoma cell lines were transduced and selected with the appropriate antibiotic (1 µg/ml puromycin or 10 µg/ml blasticidine) until the control cells died. Cells were seeded in phenol-red-free medium, synchronized by medium change and supplemented with 250 µM D-Luciferin (Bio-Rad laboratories, Hercules, CA, USA) and optionally indicated treatments. Bioluminescence was recorded for at least 4 days using the Lumicycle Device (Actimetrics, Wilmette, IL, USA). Circadian parameters were evaluated from 24 h average detrended curves using the Chronostar 3 software and visualized in GraphPad Prism 10.4.1.

Time-course gene expression analysis of selected core-clock genes was performed by qRT-PCR as described previously^106^. Briefly, 3 $\times$10^4^ cells/well were seeded in 24-well plates, allowed to adhere for 24 h and synchronized by medium change. Cells were harvested at indicated timepoints (every 3 h for 54 h) and RNA was extracted using the RNeasy Plus Mini kit (Qiagen, Hilden, Germany) according to the manufacturer. One microgram of RNA was reverse transcribed to cDNA and qRT-PCR was performed using QuantiTect Primers (Qiagen, Hilden, Germany): *GAPDH*: QT00079247; *BMAL1*: QT00011844; *PER2*: QT00011207; *NR1D1*: QT00000413; *CRY1*: QT00025067, *CRY2*: QT00094920 and *NR1D2*: QT00008897. CT values were normalized to *GAPDH* and mean gene expression to obtain fold-change values. Best-fit period values were determined using the R package DiscoRhythm based on *PER2* and *BMAL1* curves. Cosinor analysis was performed to obtain *p*-values of rhythmicity and circadian parameters (amplitude, acrophase). Data were visualized in GraphPad Prism 10.4.1. All measurements were performed in 2–3 independent biological replicates, including 3 technical replicates each. One-way ANOVA was used for comparisons between groups.

### Mechanistic modeling of TMZ chronopharmacology

Cell population behavior under TMZ treatment was simulated using a modified growth model with Allee effect. Classical model equation was expanded by a first-order decay term to account for toxic effects of alkylated DNA (DNA_A_) adducts:

1 $$\frac{{dN}}{{dt}}={C}_{{KO}}{k}_{p}N\left(1-\frac{N}{{K}_{c}}\right)\left(\frac{N}{{N}_{c}}-1\right)-{k}_{t}\left[{{\rm{DNA}}}_{{\rm{A}}}\right]$$

where *N* is cell population size, *k*_*p*_ is the populations’ proliferation rate constant, *K*_*c*_ is the environmental carrying capacity, *N*_*c*_ is the critical cell population size, *k*_*t*_ is the toxicity induced population decay rate constant, and [DNA_A_] is the concentration of alkylated DNA adduct. The factor *C*_*KO*_ was introduced to account for minor losses in cell fitness due to the introduction of gene knock-outs (wild-type cell lines *C*_*KO*_ = 1; knock-out cell lines *C*_*KO*_ = 0.85).

Cellular uptake, intracellular modifications, accumulation of methyl-diazonium ion (CH_3_N_2_^+^), and DNA alkylation were simulated using the model presented by Stéphanou et al.^109^. Constant intra- and extra-cellular pH of 7.4 was assumed over the simulation time course. Extra-cellular volume was set to 1 mL to account for in vitro experimental settings. The detoxifying reaction catalyzed by O6-methylguanin-DNA-methyltransferase (MGMT) was simulated using the model presented by Zang et al.^110^.

To model circadian fluctuations in MGMT^111^ expression, we implemented a core-clock network model previously developed by our group^18^. Connection between TMZ effect- and core-clock network model was established via the CRY component of the latter by adjusting the term for MGMT:DNA_A_ complex formation for the MGMT concentration to follow the current-to-mean CRY level-ratio in the model equation describing DNA_A_ formation. The inverse of this term was introduced for *BMAL* to model its anti-phasic relation to MGMT expression:

2 $$\frac{d\left[{{\rm{DNA}}}_{{\rm{A}}}\right]}{{dt}}={k}_{a}\left[{{\rm{CH}}}_{3}{{\rm{N}}}_{2}^{+}\right]+{k}_{d}\left[{\rm{MGMT}}:{{\rm{DNA}}}_{{\rm{A}}}\right]-{k}_{f}\frac{{\left[{\rm{CRY}}\right]}_{t}}{{\left[{\rm{CRY}}\right]}_{{mean}}}\frac{{\left[{\rm{BMAL}}\right]}_{{mean}}}{{\left[{\rm{BMAL}}\right]}_{t}}\left[{\rm{MGMT}}\right]\left[{{\rm{DNA}}}_{{\rm{A}}}\right]$$

Here, *k*_*a*_ is the alkylation reaction rate constant, *k*_*d*_ is the MGMT:DNA_A_ complex dissociation rate constant, *k*_*f*_ is the MGMT:DNA_A_ complex formation rate constant, [CH_3_N_2_^+^] is the methyl-diazonium ion concentration, [MGMT:DNA_A_] is the MGMT:DNA_A_ complex concentration, [CRY]_*t*_ is the current CRY level, [CRY]_*mean*_ is the mean CRY level over simulation time (equivalent for *BMAL* terms), [MGMT] is MGMT concentration, and [DNA_A_] is the concentration of alkylated DNA adduct. The complete set of model equations, in addition to the ones in the core-clock network model previously developed by our group^18^ is listed below. Variables and parameters are as here specified and as previously defined in the published used models by our group^18^ (a model from our group with the core-clock can be found at biomodels https://www.ebi.ac.uk/biomodels/MODEL2109140001), and by Zang et al.^110^ for MGMT, and also Stéphanou et al.^109^ for DNA alkylation.

*Pharmacokinetic and dynamic model*

Extracellular TMZ concentration:

3 $$\frac{d{T}_{o}}{{dt}}=-\frac{{p}_{T}}{{V}_{o}}{T}_{o}$$

With *p*_*T*_ being the inwards cellular transport rate, *Vo* being the extracellular volume, and *To* being the extracellular TMZ concentration.

Intracellular TMZ concentration:

4 $$\frac{{{dT}}_{i}}{{dt}}=-\left(\frac{{p}_{T2}}{{V}_{i}}+{k}_{T}\right){T}_{i}+\frac{{p}_{T}}{{V}_{i}}{T}_{o}$$

With *p*_*T2*_ being the outward cellular transport rate, *V*_*i*_ being the intracellular volume, *k*_*T*_ being the MTIC formation rate, and *T*_*i*_ being the intracellular TMZ concentration. Note that - deviating from Stéphanou et al. - rate constants were not considered as a function of pH assuming a constant pH of 7.4 over the course of simulation.

MTIC formation:

5 $$\frac{{{dM}}_{i}}{{dt}}={k}_{T}{T}_{i}-{k}_{M}{M}_{i}$$

With *k*_*M*_ being the methyl-diazonium ion formation rate, and *M*_*i*_ being the intracellular MTIC concentration.

Methyl-diazonium ion formation:

6 $$\frac{{{dC}}_{i}}{{dt}}={k}_{M}{M}_{i}-\left({k}_{{cat}}+{k}_{{add}}\right){C}_{i}$$

With *k*_*cat*_ being the methyl-diazonium ion, *k*_*add*_ being the DNA adduct formation rate, and *C*_*i*_ being the intracellular methyl-diazonium ion concentration.

DNA adduct formation:

7 $$\frac{d\left[{{\rm{DNA}}}_{{\rm{A}}}\right]}{{dt}}={k}_{a}\left[{{\rm{CH}}}_{3}{{\rm{N}}}_{2}^{+}\right]+{k}_{d}\left[{\rm{MGMT}}:{{\rm{DNA}}}_{{\rm{A}}}\right]-{k}_{f1}\frac{{\left[{\rm{CRY}}\right]}_{t}}{{\left[{\rm{CRY}}\right]}_{{mean}}}\frac{{\left[{\rm{BMAL}}\right]}_{{mean}}}{{\left[{\rm{BMAL}}\right]}_{t}}\left[{\rm{MGMT}}\right]\left[{{\rm{DNA}}}_{{\rm{A}}}\right]$$

*MGMT catalyzed detoxification*

DNA adduct-MGMT associate formation:

8 $$\frac{d{{MGMT}}_{1}}{{dt}}={k}_{f1}\frac{{\left[{\rm{CRY}}\right]}_{t}}{{\left[{\rm{CRY}}\right]}_{{mean}}}\frac{{\left[{\rm{BMAL}}\right]}_{{mean}}}{{\left[{\rm{BMAL}}\right]}_{t}}\left[{\rm{MGMT}}\right]\left[{{\rm{DNA}}}_{{\rm{A}}}\right]-{k}_{r1}{{MGMT}}_{1}-{k}_{f2}{{MGMT}}_{1}+{k}_{r2}{{MGMT}}_{2}$$

With *k*_*f1*_ being the DNA adduct-MGMT associate formation rate, *k*_*r1*_ being the DNA adduct-MGMT associate dissociation rate, *k*_*f2*_ being the DNA adduct-MGMT complex formation rate, *k*_*r2*_ being the DNA adduct-MGMT complex dissociation rate, *MGMT*_*1*_ being the DNA adduct-MGMT associate, and *MGMT*_*2*_ being DNA adduct-MGMT complex.

DNA adduct-MGMT complex formation:

9 $$\frac{{{dMGMT}}_{2}}{{dt}}={k}_{f2}{{MGMT}}_{1}-{k}_{r2}{{MGMT}}_{2}-{k}_{f3}{{MGMT}}_{2}$$

With *k*_*f3*_ being the DNA diacylation rate constant.

DNA de-acylation:

10 $$\frac{{{dMGMT}}_{3}}{{dt}}={k}_{f3}{{MGMT}}_{2}$$

With *k*_*f3*_ being the DNA diacylation rate constant.

*Cell population model*

Cell-proliferation:

11 $$\frac{{dN}}{{dt}}={C}_{{KO}}{k}_{p}N\left(1-\frac{N}{{K}_{c}}\right)\left(\frac{N}{{N}_{c}}-1\right)-{k}_{t}\left[{{\rm{DNA}}}_{{\rm{A}}}\right]$$

### Time-dependent treatment assays

Cells were seeded at a concentration of 2.5 $\times$10^3^/well and allowed to adhere for 24 h. Cells were synchronized by medium change for 0, 5, 10, 15 or 20 h and treated with the indicated treatments or treatment combinations of TMZ, KL001 or SR9011 at a pre-determined IC_50_ concentration. Cell confluence was measured every 2 h using the IncuCyte S5 (Sartorius, Göttingen, Germany). Cell confluency was normalized to cell confluency at *t* = 0 and AUC values were determined using GraphPad Prism 10.4.1. and normalized to the respective AUC values of DMSO-treated cells for each plate. All measurements were performed in 2–3 independent biological replicates, including 3 technical replicates each. One-way ANOVA was used for comparisons between groups.

## Supplementary information

Supplementary information

## Data Availability

All data produced in this study are included in the main article and the supplementary information. Raw data are available from the corresponding author upon reasonable request.
