# Supplementary material for: Personalized chronotherapy in glioblastoma: integrating circadian profiling and PK–PD modelling to optimize temozolomide timing
Source: NPJ Precis Oncol. 2025 Dec 12;10:17. doi: 10.1038/s41698-025-01205-z (PMC12796165; doi:10.1038/s41698-025-01205-z)
Supplement: Supplementary file 1 — Supplementary information [file 41698_2025_1205_MOESM1_ESM.pdf]

Supplementary Information for:

Personalized Chronotherapy in Glioblastoma: Integrating Circadian Profiling and PK–PD Modelling to Optimize Temozolomide Timing

Nina Nelson[1], Oliver Zimmer[1], Angela Relógio[1,2]

Affiliations

[1]Institute for Systems Medicine and Faculty of Human Medicine, MSH Medical School Hamburg, Hamburg, 20457, Germany

[2]Corresponding author

Angela Relógio, angela.relogio@medicalschool-hamburg.de

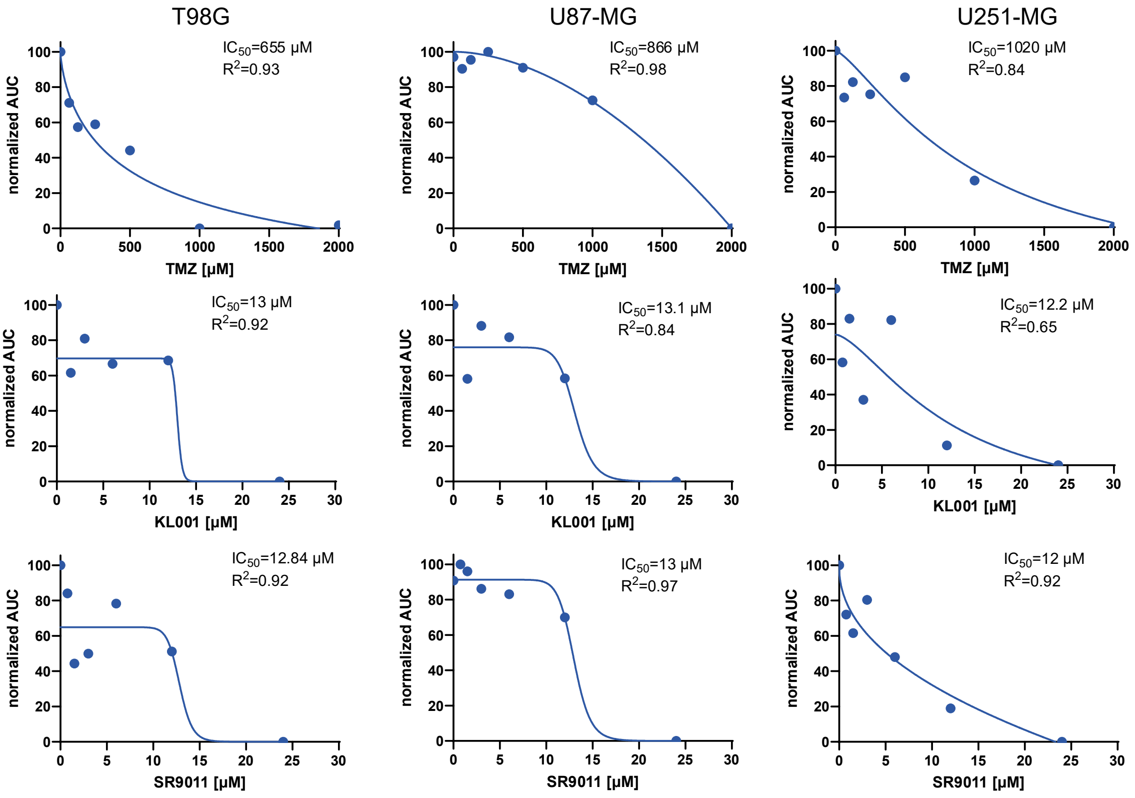

Supplementary Figure 1: IC_{50} values of the drugs used in this study. *IC_{50} values of DMSO normalized AUC values after 30h of treatment were calculated using the function [inhibitor] vs. response-variable slope (four parameters) in GraphPad Prism. Points represent mean values of 5 replicates.*

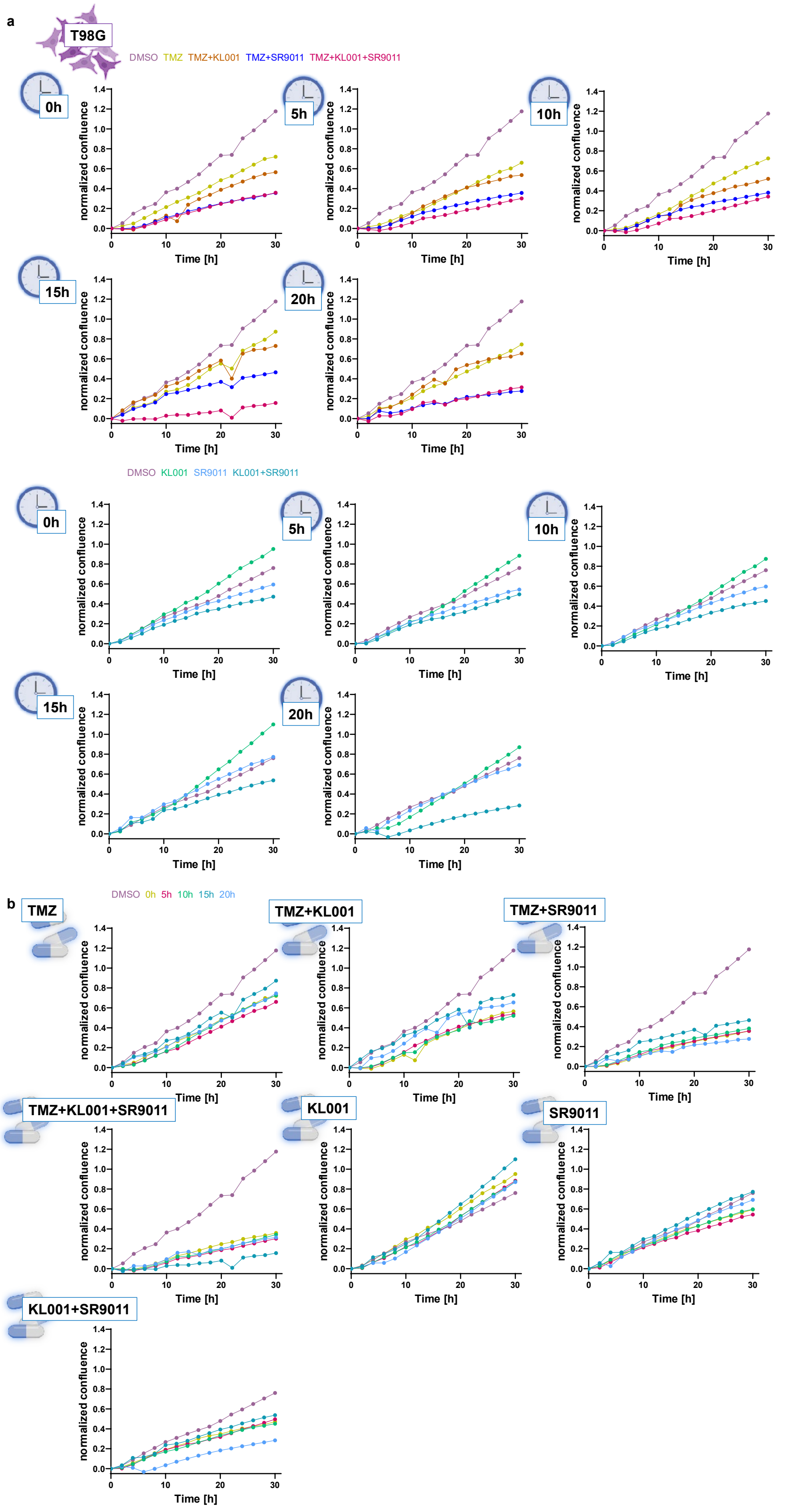

Supplementary Figure 2: Proliferation curves of T98G cells treated with different drugs and drug combinations at different timepoints after synchronization. *Data points represent means of three biological replicates with three technical replicates each. Icons were generated in Biorender.com.*

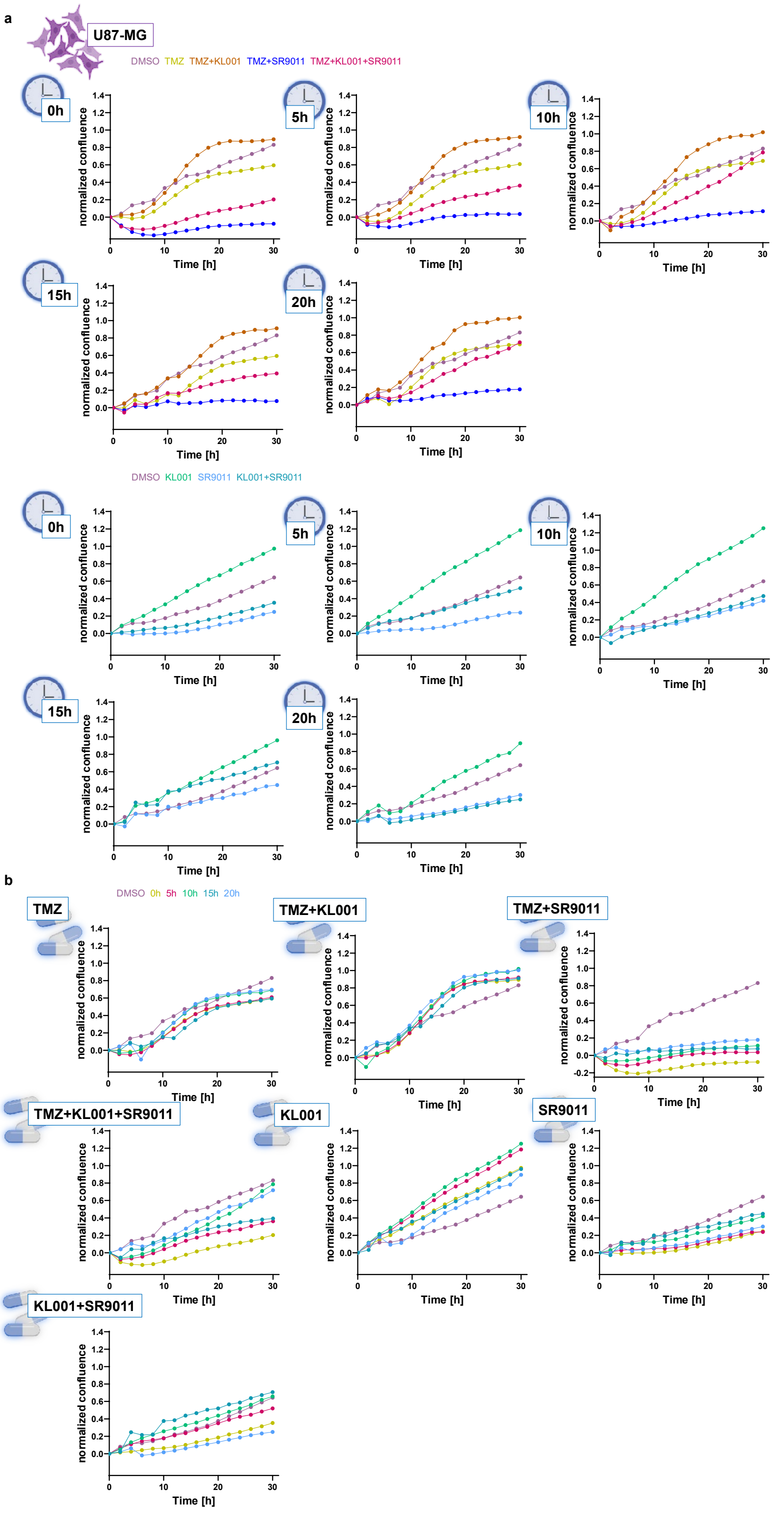

Supplementary Figure 3: Proliferation curves of U87-MG cells treated with different drugs and drug combinations at different timepoints after synchronization. *Data points represent means of three biological replicates with three technical replicates each. Icons were generated in Biorender.com.*

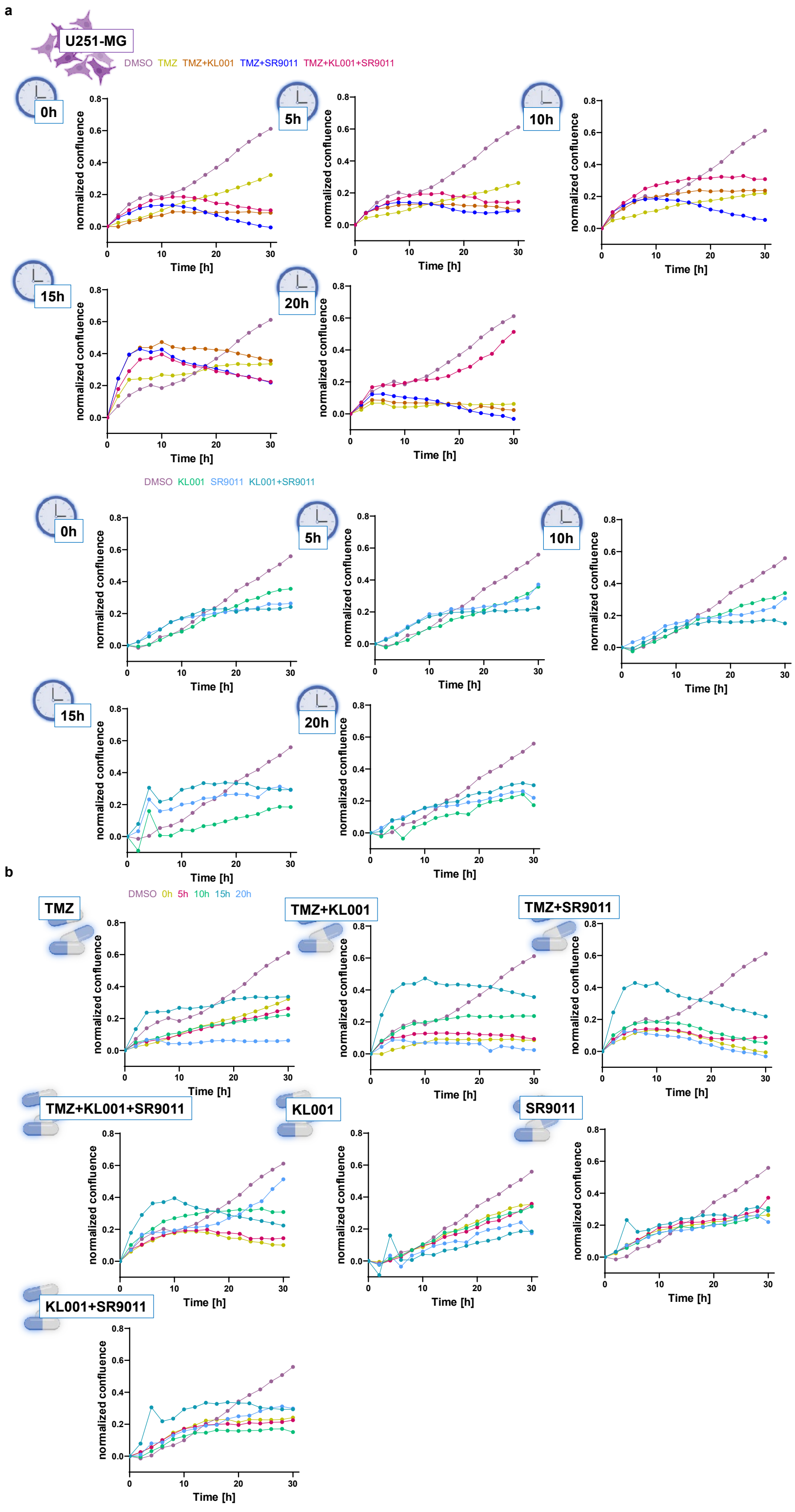

Supplementary Figure 4: Proliferation curves of U251-MG cells treated with different drugs and drug combinations at different timepoints after synchronization. *Data points represent means of three biological replicates with three technical replicates each. Icons were generated in Biorender.com.*

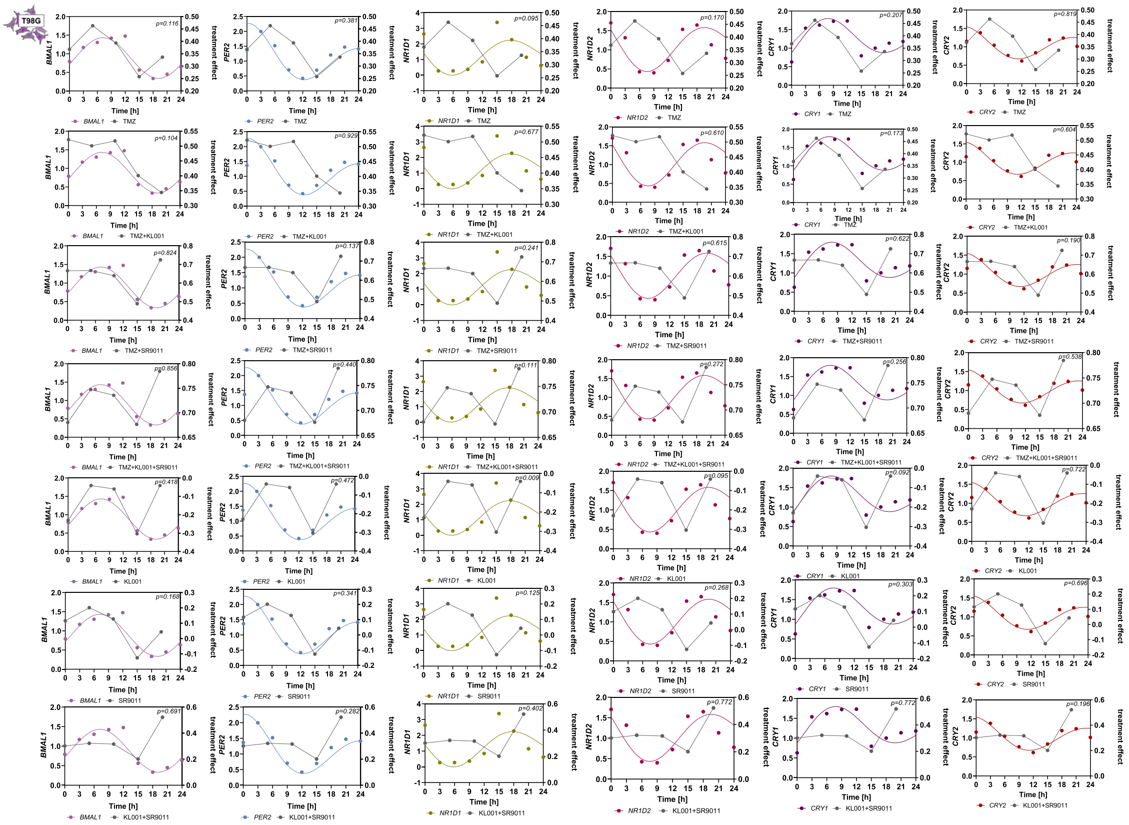

Supplementary Figure 5: Correlation of gene expression with treatment effects in T98G WT. *Treatment effects were correlated with the gene expression data obtained by q-RT-PCR using Pearson correlation. All data were obtained from three independent biological replicates each containing three technical replicates. Icons were generated in Biorender.com.*

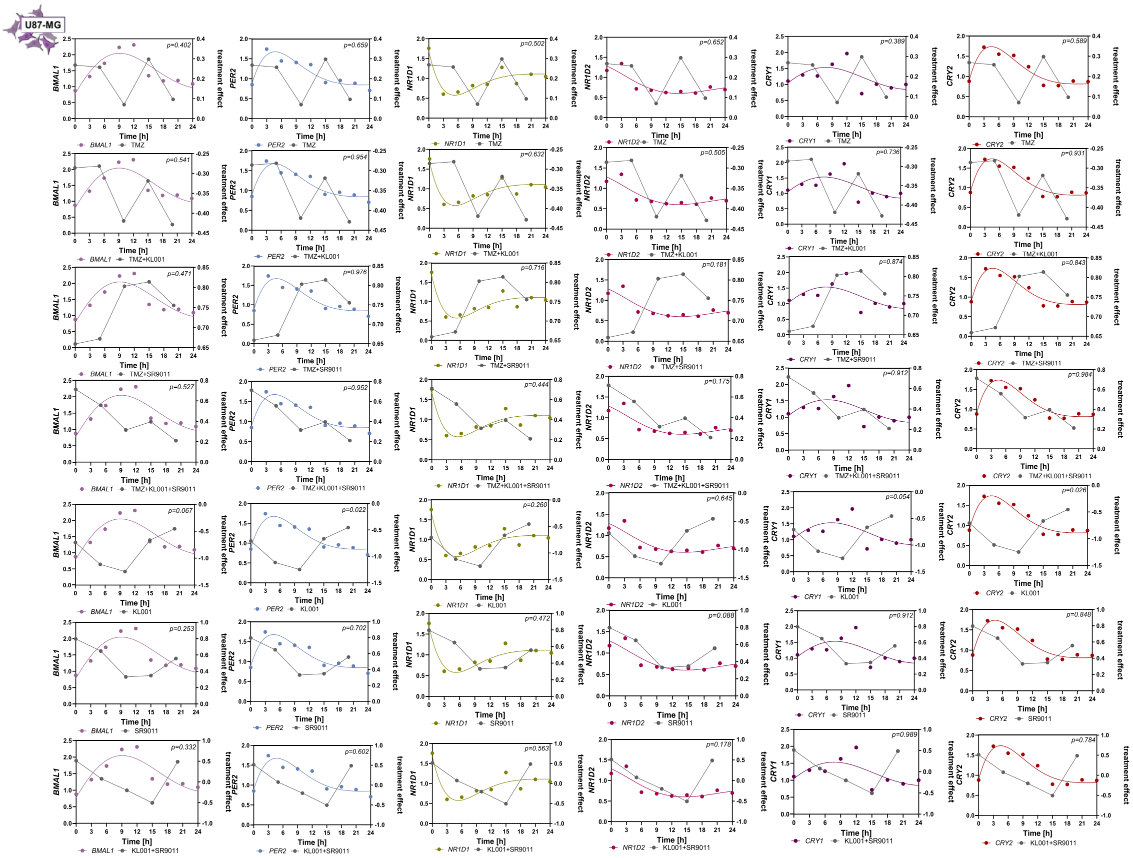

Supplementary Figure 6: Correlation of gene expression with treatment effects in U87-MG WT. *Treatment effects were correlated with the gene expression data obtained by q-RT-PCR using Pearson correlation. All data were obtained from three independent biological replicates each containing three technical replicates. Icons were generated in Biorender.com.*

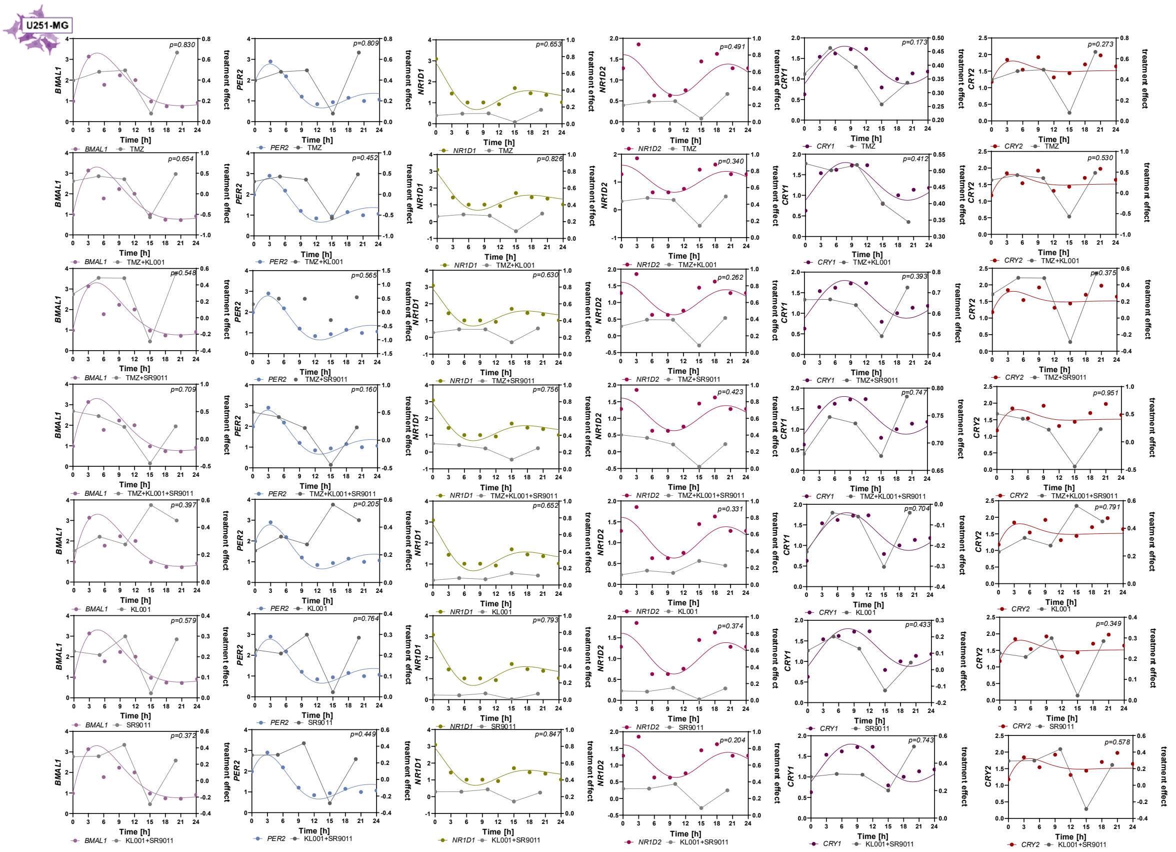

Supplementary Figure 7: Correlation of gene expression with treatment effects in U251-MG WT. *Treatment effects were correlated with the gene expression data obtained by q-RT-PCR using Pearson correlation. All data were obtained from three independent biological replicates each containing three technical replicates. Icons were generated in Biorender.com.*

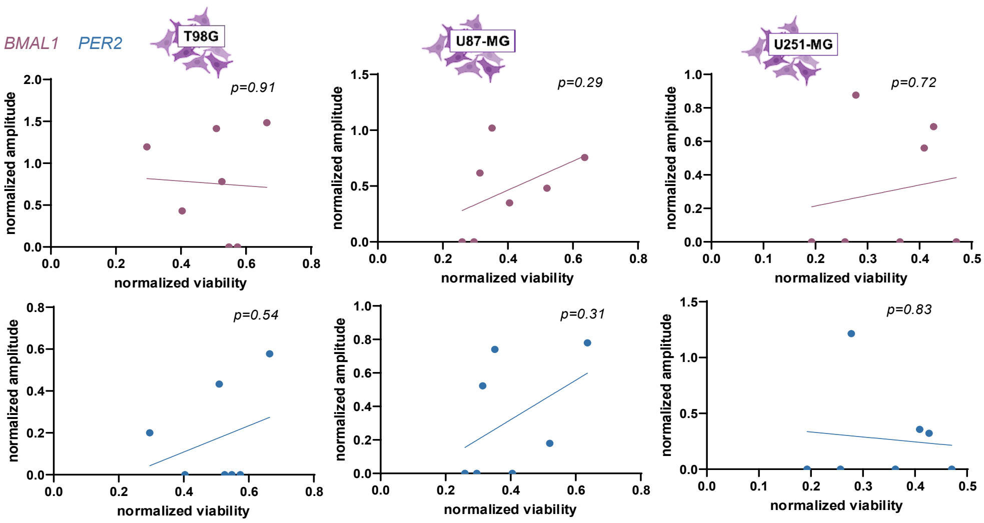

Supplementary Figure 8: Pearson correlation between normalized viability and normalized Lumicycle amplitude under treatment. *Data acquisition was performed at least twice with 3 replicates.*

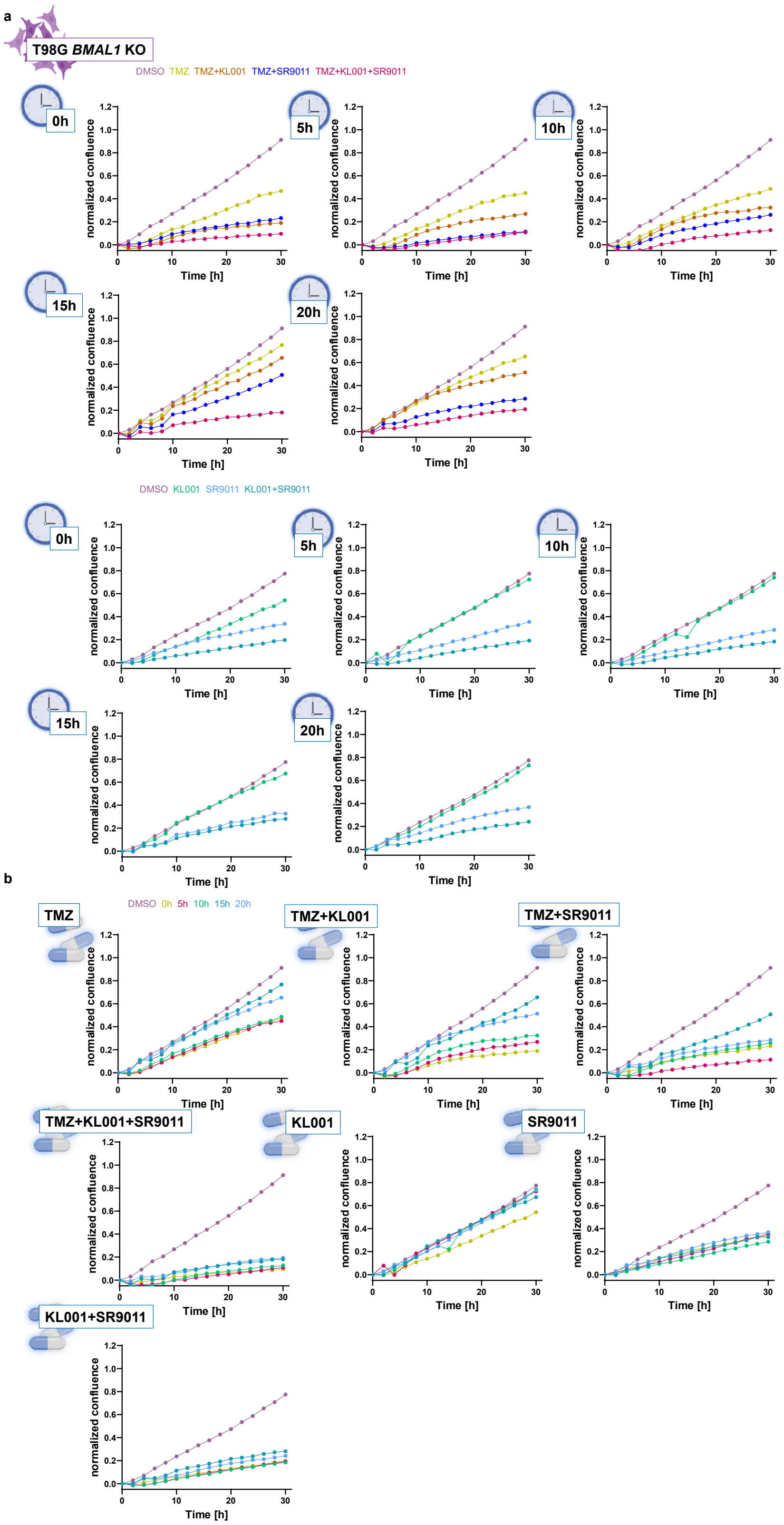

Supplementary Figure 9: Proliferation curves of T98G BMAL1 KO cells treated with different drugs and drug combinations at different timepoints after synchronization. *Data points represent means of three biological replicates with three technical replicates each. Icons were generated in Biorender.com.*

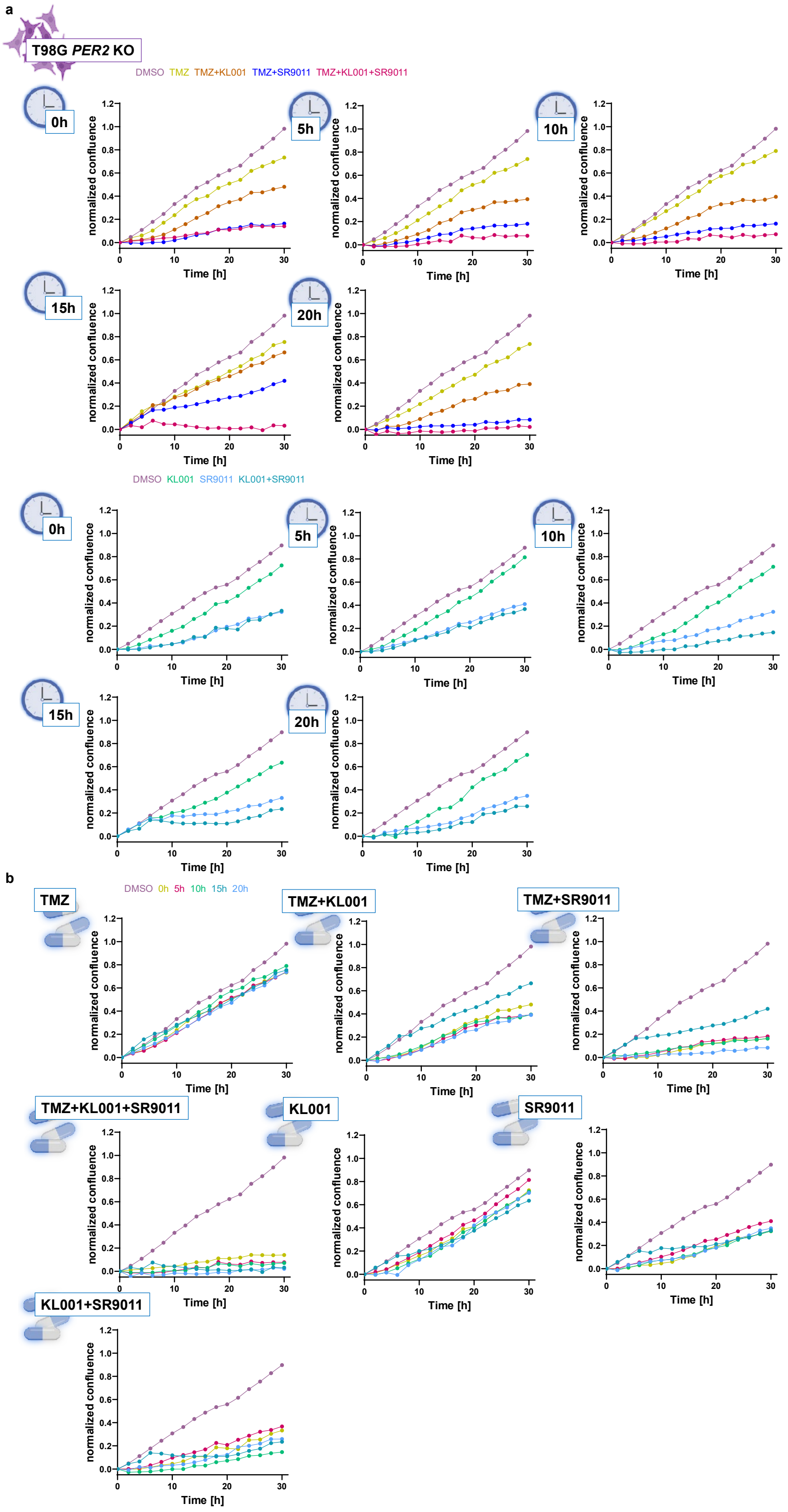

Supplementary Figure 10: Proliferation curves of T98G PER2 KO cells treated with different drugs and drug combinations at different timepoints after synchronization. *Data points represent means of three biological replicates with three technical replicates each. Icons were generated in Biorender.com.*

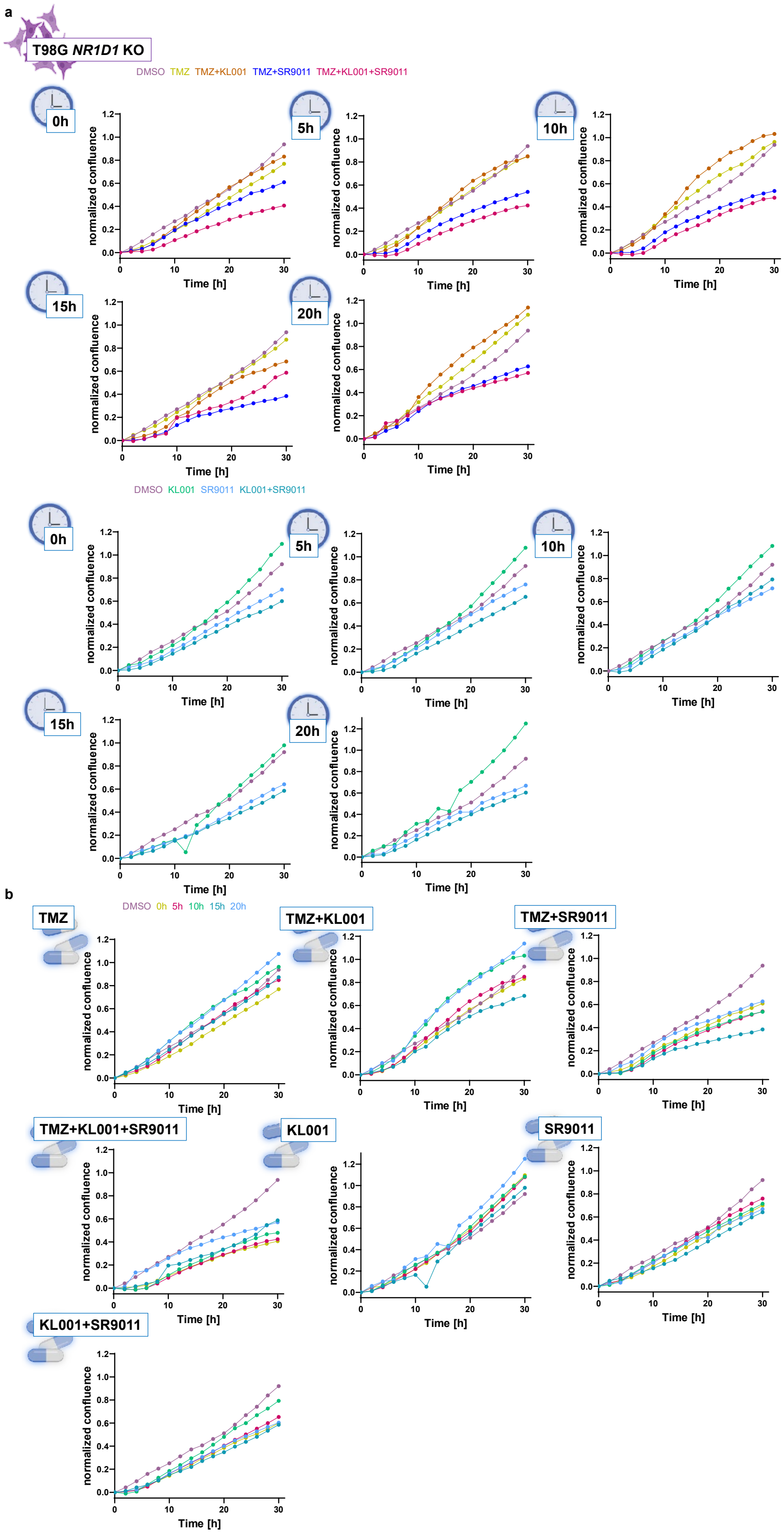

Supplementary Figure 11: Proliferation curves of T98G NR1D1 KO cells treated with different drugs and drug combinations at different timepoints after synchronization. *Data points represent means of three biological replicates with three technical replicates each. Icons were generated in Biorender.com.*

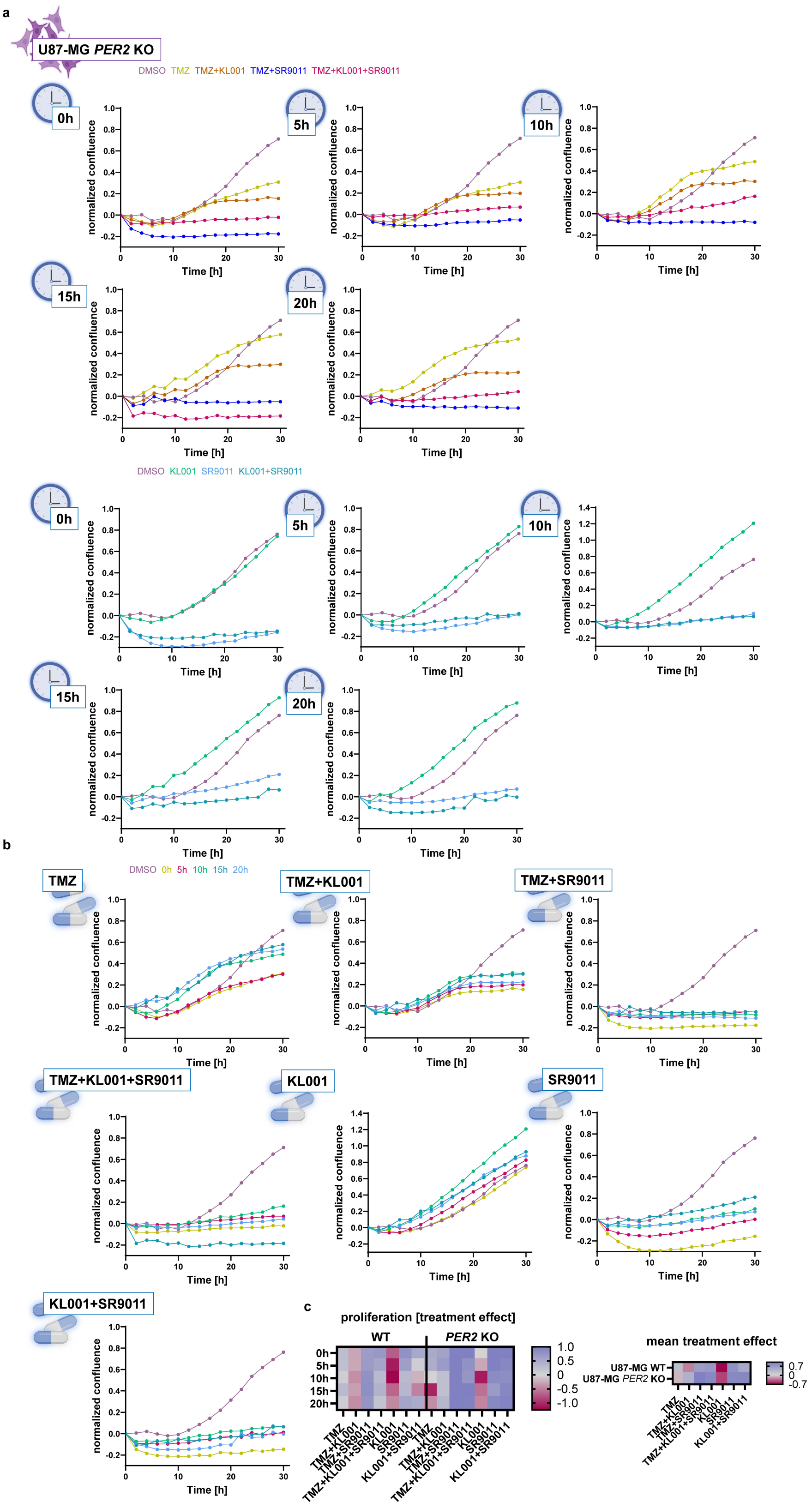

Supplementary Figure 12: Proliferation curves of U87-MG PER2 KO cells treated with different drugs and drug combinations at different timepoints after synchronization*. Data points represent means of three biological replicates with three technical replicates each. Icons were generated in Biorender.com.*

Supplementary Table 1: Treatment effects. *Listed are the mean and standard deviations for all treatments. Measurements were performed over three biological replicates with three technical replicates each.*

TREATMENT	T98G		U87-MG		U251-MG	
	MEAN	SD	MEAN	SD	MEAN	SD
TMZ 0h	0,37	0,12	0,27	0,12	0,40	0,38
TMZ 5h	0,46	0,12	0,26	0,12	0,48	0,29
TMZ 10h	0,39	0,10	0,07	0,16	0,49	0,35
TMZ 15h	0,26	0,09	0,30	0,12	0,08	0,61
TMZ 20h	0,34	0,14	0,10	0,09	0,67	0,21
TMZ+KL001 0h	0,52	0,10	-0,29	0,48	0,31	0,53
TMZ+KL001 5h	0,50	0,14	-0,28	0,39	0,43	0,37
TMZ+KL001 10h	0,52	0,21	-0,42	0,62	0,36	0,35
TMZ+KL001 15h	0,40	0,15	-0,32	0,65	-0,57	0,97
TMZ+KL001 20h	0,34	0,15	-0,43	0,38	0,48	0,47
TMZ+SR9011 0h	0,67	0,11	0,66	0,29	0,29	0,70
TMZ+SR9011 5h	0,67	0,11	0,67	0,19	0,49	0,37
TMZ+SR9011 10h	0,64	0,07	0,80	0,04	0,48	0,35
TMZ+SR9011 15h	0,49	0,14	0,81	0,12	-0,29	0,89
TMZ+SR9011 20h	0,73	0,11	0,76	0,09	0,54	0,36
TMZ+KL001+SR9011 0h	0,68	0,14	0,71	0,12	0,51	0,38
TMZ+KL001+SR9011 5h	0,75	0,13	0,56	0,24	0,42	0,33
TMZ+KL001+SR9011 10h	0,74	0,10	0,31	0,25	0,22	0,58
TMZ+KL001+SR9011 15h	0,68	0,14	0,40	0,40	-0,45	1,16
TMZ+KL001+SR9011 20h	0,78	0,12	0,21	0,56	0,23	0,64
KL001 0h	-0,23	0,36	-0,71	0,31	0,23	0,27
KL001 5h	-0,04	0,19	-1,12	0,42	0,33	0,24
KL001 10h	-0,06	0,24	-1,25	0,32	0,28	0,21
KL001 15h	-0,30	0,32	-0,67	0,34	0,56	0,19
KL001 20h	-0,04	0,38	-0,45	1,02	0,45	0,24
SR9011 0h	0,12	0,29	0,80	0,07	0,23	0,30
SR9011 5h	0,20	0,23	0,65	0,22	0,21	0,21
SR9011 10h	0,13	0,16	0,33	0,17	0,30	0,33
SR9011 15h	-0,13	0,29	0,35	0,07	0,02	0,33
SR9011 20h	0,04	0,36	0,56	0,35	0,28	0,37
KL001+SR9011 0h	0,30	0,17	0,51	0,21	0,29	0,04
KL001+SR9011 5h	0,32	0,14	0,08	0,39	0,30	0,14
KL001+SR9011 10h	0,32	0,14	-0,20	0,51	0,44	0,24
KL001+SR9011 15h	0,20	0,10	-0,51	0,97	-0,29	0,66
KL001+SR9011 20h	0,52	0,17	0,49	0,26	0,25	0,56

Supplementary Table 2: Correlation of treatment effects and gene expression. *Shown are the p-values and Pearson r values. Measurements were performed over three biological replicates with three technical replicates each.*

T98G

p-values

GENE	TMZ	TMZ+KL001	TMZ+SR9011	TMZ+KL001+SR9011	KL001	SR9011	KL001+SR9011
BMAL1	0,116	0,104	0,824	0,856	0,418	0,168	0,691
PER2	0,381	0,929	0,137	0,440	0,472	0,341	0,282
NR1D1	0,095	0,677	0,241	0,111	0,009	0,125	0,402
NR1D2	0,170	0,610	0,615	0,272	0,095	0,268	0,772
CRY1	0,207	0,653	0,622	0,256	0,092	0,303	0,745
CRY2	0,819	0,604	0,190	0,538	0,722	0,696	0,196

Pearson r

GENE	TMZ	TMZ+KL001	TMZ+SR9011	TMZ+KL001+SR9011	KL001	SR9011	KL001+SR9011
BMAL1	0,784	0,800	0,138	0,113	0,476	0,722	-0,245
PER2	0,509	-0,056	0,759	0,456	0,428	0,546	0,603
NR1D1	-0,813	-0,257	-0,644	-0,791	-0,962	-0,774	-0,490
NR1D2	-0,720	-0,312	-0,307	-0,612	-0,812	-0,617	-0,180
CRY1	0,679	0,276	0,302	0,629	0,816	0,582	0,202
CRY2	0,143	-0,316	0,698	0,371	0,220	0,241	0,691

U87-MG

p-values

GENE	TMZ	TMZ+KL001	TMZ+SR9011	TMZ+KL001+SR9011	KL001	SR9011	KL001+SR9011
BMAL1	0,402	0,541	0,471	0,527	0,067	0,253	0,332
PER2	0,659	0,954	0,976	0,952	0,022	0,702	0,602
NR1D1	0,502	0,632	0,716	0,444	0,260	0,472	0,563
NR1D2	0,652	0,505	0,181	0,175	0,645	0,088	0,178
CRY1	0,389	0,736	0,874	0,912	0,054	0,912	0,989
CRY2	0,589	0,931	0,843	0,984	0,026	0,848	0,784

Pearson r

GENE	TMZ	TMZ+KL001	TMZ+SR9011	TMZ+KL001+SR9011	KL001	SR9011	KL001+SR9011
BMAL1	-0,490	-0,369	0,429	-0,381	-0,851	-0,632	-0,554
PER2	-0,271	-0,036	-0,019	-0,037	-0,930	-0,237	-0,318
NR1D1	0,402	0,293	-0,225	0,453	0,625	0,428	0,350
NR1D2	0,277	0,400	-0,708	0,715	0,283	0,821	0,712
CRY1	-0,501	-0,209	-0,099	0,069	-0,873	-0,069	-0,009
CRY2	-0,328	-0,054	-0,124	0,013	-0,922	-0,120	-0,170

U251-MG

p-values

GENE	TMZ	TMZ+KL001	TMZ+SR9011	TMZ+KL001+SR9011	KL001	SR9011	KL001+SR9011
BMAL1	0,830	0,654	0,548	0,709	0,397	0,579	0,372
PER2	0,809	0,452	0,565	0,160	0,205	0,764	0,449
NR1D1	0,653	0,826	0,630	0,756	0,652	0,793	0,847
NR1D2	0,491	0,340	0,262	0,423	0,331	0,374	0,204
CRY1	0,173	0,412	0,393	0,747	0,704	0,433	0,743
CRY2	0,273	0,530	0,375	0,951	0,791	0,349	0,578

Pearson r

GENE	TMZ	TMZ+KL001	TMZ+SR9011	TMZ+KL001+SR9011	KL001	SR9011	KL001+SR9011
BMAL1	0,134	0,275	0,363	0,231	-0,494	0,337	0,517

PER2	0,150	0,446	0,348	0,732	-0,682	0,186	0,448
NR1D1	-0,276	-0,137	-0,295	0,193	-0,277	-0,163	-0,121
NR1D2	-0,412	-0,547	-0,623	-0,471	0,555	-0,516	-0,683
CRY1	0,717	0,481	0,498	0,200	0,234	0,463	0,203
CRY2	0,612	0,378	0,515	-0,039	0,165	0,539	0,338
